# Resumption of Menses and Normalisation of Body Weight in Adolescents Undergoing Specialised Treatment for Anorexia Nervosa Inpatient or Outpatient Treatment

**DOI:** 10.1002/erv.70139

**Published:** 2026-06-18

**Authors:** Annika Woltmann, Linus Imken, Vivien Kaiser, Johanna L. Keeler, Daniel Le Grange, Christoph U. Correll, Verena Haas

**Affiliations:** ^1^ Department of Child and Adolescent Psychiatry Charité—Universitätsmedizin Berlin Corporate Member of Freie Universität Berlin and Humboldt Universität zu Berlin Berlin Germany; ^2^ Centre for Research in Eating and Weight Disorders Department of Psychological Medicine Institute of Psychiatry, Psychology and Neuroscience King's College London London UK; ^3^ Department of Child and Adolescent Psychiatry, Psychosomatics and Psychotherapy LVR University Hospital Essen University of Duisburg‐Essen Essen Germany; ^4^ Department of Psychiatry and Behavioral Sciences University of California San Francisco California USA; ^5^ Department of Psychiatry and Behavioral Neurosciences The University of Chicago Chicago Illinois USA; ^6^ Charité—Universitätsmedizin Berlin Corporate Member of Freie Universität Berlin and Humboldt Universität zu Berlin Berlin Germany; ^7^ German Center for Mental Health (DZPG) Berlin Germany; ^8^ German Center for Child and Adolescent Health (DZKJ) Berlin Germany; ^9^ Department of Psychiatry Research The Zucker Hillside Hospital Glen Oaks New York USA; ^10^ Department of Psychiatry and Molecular Medicine The Zucker School of Medicine at Hofstra/Northwell Hempstead New York USA

**Keywords:** amenorrhoea, anorexia nervosa, predictors, resumption of menses

## Abstract

**Objective:**

To predict resumption of menses (ROM) in patients with anorexia nervosa (AN).

**Method:**

Females aged 9–17 years with AN, receiving inpatient multimodal treatment (IMT) or family‐based treatment (FBT) underwent assessments at baseline, 6‐month and 12‐month follow‐up (12FU). A menstrual questionnaire recorded date, weight and height at loss of menses (LOM) and ROM occurring up to 30 months after 12FU. A stepwise backward logistic regression analysis evaluated predictors for menstruation at 12FU.

**Results:**

Among 99 females (age = 14.8 ± 1.7) with AN (restrictive = 85 [86%], binge‐purge = 8 [8%], atypical = 6 [6%]), and percent median body mass index (%median BMI) = 78.9 [IQR = 74.3, 82.8] undergoing IMT (*n* = 50) or FBT (*n* = 49), 66 (66.7%) had at baseline secondary amenorrhoea, 26 (26.3%) were without menarche, 5 (5.1%) had regular menses, and 2 (2.0%) used contraceptives. At 12FU, menses were present in 45 (45.5%) patients and associated with baseline menarche (OR = 6.04, *p* = 0.002) and higher 12FU %mBMI (OR = 1.08, *p* = 0.006) (*R* ^2^ = 0.251). After an average of 15 months of amenorrhoea [IQR = 12.0, 25.0] (*n* = 41 with information), %mBMI at ROM (92.0 [IQR = 86.6, 97.8], equalling BMI percentile = 25.0; range = 1–60), was 6.8 %mBMI units > LOM (85.2 [IQR = 79.9, 92.0]; *p* = 0.001), and 6.5 %mBMI units < premorbid %mBMI (98.5 [IQR = 94.1109.7]; *p* < 0.001).

**Conclusions:**

ROM follows weight recovery. Full restoration to premorbid BMI percentile is not necessary for ROM.

## Highlights

- The prevalence of regular menses in young patients with anorexia nervosa, receiving inpatient multimodal treatment (IMT) or family‐based treatment (FBT), improved from 5% at baseline to 45% at 12‐month follow‐up (12FU).
- The presence of menarche at baseline and a higher percent median body mass index (BMI) at 12FU were associated with regular menses at 12FU while eating disorder‐specific psychopathology, compulsive exercise, treatment duration, medication, psychiatric comorbidities and treatment approach (IMT vs. FBT) had no significant influence. [Correction added on 21 August 2026, after first online publication: The word “percent” has been added before “median body mass index”.]
- The median BMI percentile at resumption of menses was 25 (range = 1–60), which was significantly higher than the BMI percentile at loss of menses, but significantly lower than the premorbid BMI percentile.

## 1 Introduction

Anorexia nervosa (AN) is a severe and often protracted disorder characterised by intentional weight loss and distorted body image. The lifetime prevalence of AN is up to 3.6% in females and 0.3% in males worldwide (Galmiche et al. 2019) with a peak onset at age 15.5 years (Solmi et al. 2022). One of the physical symptoms in postmenarchal females is absence of menses, known as secondary amenorrhoea, which is primarily caused by endocrine dysfunction resulting from hypothalamic suppression of the hypothalamic‐pituitary‐ovarian axis in response to an energy deficit (Haines 2023; Karountzos et al. 2017). The classification of menstrual status in young females with AN can be challenging. As menarche typically occurs between 10 and 15 years of age (Markevych et al. 2022), in premenarchal girls with AN, the physiological term primary amenorrhoea is used if menarche has not occurred by the age of 15 or 3 years post‐thelarche (Gasner and Rehman 2025). The average age at menarche is approximately 11.9–13.0 years in Western societies (Ramraj and Subramanian 2021; Brix et al. 2019; Martinez 2020). Therefore, in females with AN < 15 years old, it is impossible to determine whether or not the absence of menarche is illness‐related.

This classification, however, is clinically relevant for evaluating whether the achieved body weight (BW) is sufficient to support both physiological and psychological recovery, as resumption of menses (ROM) is generally accepted as a marker for an individual's healthy BW (Rosen 2010; Herpertz et al. 2019). Patients without menarche have often been excluded from studies on menstrual status, although the incidence rates of AN particularly in 10‐14‐year‐old girls have increased from 1985–2019, indicating the need to expand research in young premenarchal girls with AN (van Eeden et al. 2023). Dempfle et al. 2013 reported in 2013 that 20% of girls aged 11–18 years with AN were premenarchal. Early onset and prolonged amenorrhoea are risk factors for complications of AN, including reduced bone mineral density for age, a commonly irreversible secondary sequela of AN (Legroux‐Gérot et al. 2007).

A central marker of illness severity and degree of recovery from AN is an individual's BW in relation to height, age and sex. Since absolute body mass index (BMI) varies with age in children and adolescents, age‐and‐sex‐adjusted BMI percentiles or z‐scores, and percent median BMI (%mBMI) based on a reference population, are commonly used in minors (Schaumberg 2025; Gradl‐Dietsch et al. 2024; Medical Management of Restrictive Eating Disorders in Adolescents and Young Adults 2022; Engelhardt et al. 2021). A main goal of AN treatment is medical stabilisation, particularly through physiological recovery to premorbid healthy weight trajectories. Thus, setting a healthy weight range goal is an important part of treatment, although it is a moving target, as the target weight range must be adjusted as patients grow older and taller (Jary Franklin et al. 2024; Norris et al. 2018). The healthy BW range is typically based on population norms, age and sex. While some clinicians consider individual factors, like growth curves or menstrual status, others set fixed targets (Herpertz‐Dahlmann 2021; Steinberg et al. 2023). Jary Franklin et al. 2024 calculated the target weight for adolescents with AN and atypical anorexia nervosa (AAN) using reference population norms versus individual historical growth. The within‐person variation between methods varied up to 11.3 kg for AN, and up to 19.3 kg for AAN (Jary Franklin et al. 2024). These findings highlight the complexity and potential inaccuracy of relying on standardised methods to determine individual healthy BWs in clinical practice.

German S3‐guidelines for the treatment of AN (Herpertz et al. 2019) recommend the 25th BMI percentile for adolescents as a healthy BW because previous studies suggested this as the *average* weight at ROM (Golden et al. 2008; Swenne 2004; Dempfle et al. 2013). However, a study by Golden et al. 2008 in 36 adolescent girls showed that weight at ROM ranged from the 14th‐39th BMI percentile. This finding indicates that the 25th BMI percentile is not an appropriate target weight for all patients. Since ROM is considered a meaningful biological marker of recovery, its association with other clinical parameters has been investigated to predict a healthy BW range (Dempfle et al. 2013; Castellini et al. 2020; Faust et al. 2013). Individual studies show that reaching a target weight range between the 15th‐20th BMI percentile (Dempfle et al. 2013), a shorter duration of illness (Dempfle et al. 2013; Castellini et al. 2020), binge‐purging AN subtype (Castellini et al. 2020), and menarche before admission (Dempfle et al. 2013), were all associated with a higher probability for ROM. Findings regarding disorder‐specific psychopathology, however, were contradictory (Castellini et al. 2020; Faust et al. 2013). About 65% of patients with secondary amenorrhoea at baseline had ROM within 12 months (Golden et al. 2008; Faust et al. 2013).

In Germany, patients with severe AN are usually treated as inpatients or in day‐hospital settings aiming at the 25th BMI percentile for discharge according to the S3‐guidelines (Herpertz et al. 2019). In contrast family‐based treatment (FBT), which is recommended as first‐line treatment in the guidelines of the American Psychiatric Association (Crone et al. 2023; NICE guidelines n.d.), National Institute for Health and Care Excellence (Crone et al. 2023; NICE guidelines n.d.), and Canadian (Couturier et al. 2020), Australian and New Zealand (Beumont et al. 2004) guidelines for adolescents with AN, does not define a specific target weight range, but the treatment aim is nutritional rehabilitation that leads to regular menses (Lock and Le Grange 2015). These differences in guidelines may have an influence on menstrual recovery in youth treated either with FBT or as inpatients/day‐hospital patients.

To examine predictors of ROM, including the effect of ambulatory FBT versus hospital treatment, this study aimed to investigate menstrual status over 12 months in female adolescents with AN (aged 9–17 years) who received either inpatient multimodal treatment (IMT) or FBT within a University clinic in Germany specialising in the treatment of youth with AN. Specifically, we assessed the menstrual status at baseline and 12‐month follow‐up (12FU), and identified baseline predictors of regular menses at 12FU. In a subsample of patients with secondary amenorrhoea at baseline, we further compared weight‐related parameters and time course between premorbid status, loss of menses (LOM) and ROM. We also investigated whether changes in menstrual status differed between FBT‐ and IMT‐treated patients.

Based on the available literature, we hypothesised that (1) secondary amenorrhoea would be present in 80% of patients at baseline of which 65% will have reached ROM at 12FU; (2) higher age‐and‐sex‐adjusted weight at 12FU, menarche at baseline and lower baseline eating disorder‐specific psychopathology would independently be associated with higher odds of menses at 12FU; (3) age‐and‐sex‐adjusted weight at premorbid status, LOM and ROM would be correlated with each other, and (4) FBT‐treated patients would experience a more rapid ROM compared to IMT‐treated patients even with similar weight trajectories, as FBT specifically targets ROM as a treatment aim and patients remain in their familiar social and physical environment.

## 2 Methods

### 2.1 Study Population

Patients eligible for this study included females aged 9–17 years old, diagnosed with restrictive, atypical or binge‐purge AN who were not > 3 months after baseline on oral contraceptive medications or medications known to affect sex hormone status directly or via prolactin elevation and who did not have a serious or acute physical disorder except for AN. Patients had participated either in one of two prospective, naturalistic IMT outcome studies (Nadler et al. 2022), in a prospective FBT pilot study (Haas et al. 2024) or in a naturalistic FBT outcome study and were required to have both a baseline and 12FU assessment. The responsible ethics committee approved both IMT studies (reference number ES2/051/18, EA2/240/22) and both FBT studies (EA2/133/19, EA2/063/23). All legal guardians provided written informed consent and participants provided written assent before participating in either study, which were all conducted in accordance with the Declaration of Helsinki on ‘Ethical Principles for Medical Research Involving Human Subjects’. For details about in‐ and exclusion criteria of the IMT and FBT studies, see Supplemental Methods.

### 2.2 Treatment Specifications

Patients who received IMT were treated following the German S3‐guidelines for the diagnosis and treatment of AN (Herpertz et al. 2019). IMT included high‐caloric meal plans, psychotherapy, nutritional counselling, art therapy, sports therapy, and body‐oriented therapy. Therapy sessions focused on the individual patient while family consultations took place once a week. Meals were provided by the clinic staff according to sex‐and‐age‐appropriate meal plans, which were increased if an expected rate of weekly weight gain of ≥ 500 g/week (until end of 2019) or ≥ 700 g/week (from 2020 onwards) was not met. When determining individual weight gain targets, the team considered the 25th BMI percentile as well as individual premorbid weight development.

Patients receiving FBT were treated by certified FBT‐therapists within the same clinic, and in accordance with the FBT manual (Lock and Le Grange 2015). In FBT, patients were treated as outpatients, remaining in their familiar home environment. Parents were seen as the primary resource in helping their child recover. Meals were managed by the parents, who were guided and supported by the FBT therapist. When patients were medically unstable, as defined by the Guidelines of the Society of Adolescent Health and Medicine (Golden et al. 2003), a short inpatient admission in a paediatric or child and adolescent psychiatric ward took place. These admissions served the purpose of medical stabilisation and lasted approximately 1–4 weeks. If the second attempt of FBT after discharge from medical stabilisation did not result in sufficient weight gain, based on clinical judgement, a transfer to IMT was advised (occurring in *n* = 3 of our study sample). According to the FBT manual (Lock and Le Grange 2015, 37), the treatment goal for girls is ‘nutritional rehabilitation that leads to normal menstruation rather than targeting a specific weight’. Therefore, families were not given a specific weight target. Rather, therapists focused on premorbid weight history, reduction of eating disorder‐specific psychopathology and menstrual recovery.

### 2.3 Assessments

AN was diagnosed using K‐SADS (Kiddie Schedule for Affective Disorders and Schizophrenia) (Kaufman et al. 1997) or MINI (Mini‐International Neuropsychiatric Interview) (Sheehan et al. 1998) according to Diagnostic and Statistical Manual of Mental Disorders, 5th Revision (DSM‐V) (Association 2022) or to International Statistical Classification of Diseases and Related Health Problems, 10th Revision (ICD‐10) (International statistical classification of diseases 2004). In the IMT and FBT study groups, patient characteristics were recorded at baseline (start of treatment) and after 6 and 12 months (6FU, 12FU). During IMT, patients were weighed at least weekly on a chair scale (KERN MCB 300K100 M) in the morning, fasting and in underwear. In FBT, weight was recorded before the FBT sessions by the FBT therapist or by the parents according to detailed instructions when telehealth was offered during the COVID‐19 pandemic. The following parameters of FBT‐ and IMT‐treated cohorts were assessed: date of birth, sex, AN subtype, psychiatric comorbidities (Sheehan et al. 1998), psychotropic medication, treatment duration; at baseline, 6FU and 12FU: BW, height, compulsive exercise (Compulsive Exercise Test, CET (Schlegl et al. 2022)), eating disorder‐specific psychopathology (Eating Disorder Examination Questionnaire, EDEQ (Hilbert et al. 2007)), and menstrual status (no menarche, secondary amenorrhoea, regular menses, contraceptive‐intake). Regular menses were defined as ≥ 3 consecutive cycles. The following parameters were assessed with a menstrual questionnaire that was sent out to the families after study conclusion if the data had not been collected during treatment: age, height and weight at LOM, at ROM and prior to AN onset. If ROM was not reached by 12FU, the families were contacted up to 30 months after 12FU to maximise completeness of the menstrual dataset.

The following weight metrics were assessed at all time‐points: BMI (calculated as kg/m^2^), %mBMI, BMI percentiles and z‐scores via Ped(z) Paediatrician's Calculator (Gräfe n.d.) with reference percentiles of Kromeyer‐Hauschild et al. 2001. Because the reference percentiles of Kromeyer‐Hauschild et al. 2001 apply to youth ≤ 18 years, the age of the patients was reported as 17.99 years if 12FU took place after the 18th birthday to use the same reference percentiles throughout.

### 2.4 Statistical Analysis

Statistical analyses were performed with IBM SPSS Statistics Version 24. Each parameter was tested for normal distribution using the Shapiro Wilk test. Descriptive statistics were presented as mean ± standard deviation (SD) and range (normally distributed) or median and interquartile range (IQR) (non‐normally distributed).

The difference in percentage of patients without regular menses between baseline and 12FU was calculated with the McNemar test. To identify predictors for regular menses at 12FU, patients were divided into two groups: menses at 12FU versus no menses at 12FU. Group comparisons were performed by independent samples *t*‐test (normal distribution) or Mann‐Whitney U‐test (non‐normal distribution) for continuous variables, and Chi‐square test or Fisher exact test for categorical variables. Parameters with significant group differences were included as independent variables in a multivariable stepwise backward logistic regression model which used regular menses at 12FU as the dependent variable.

To assess differences between BW parameters at ROM, LOM and premorbid BW status on an intra‐individual level, dependent samples *t*‐tests (normal distribution) or Wilcoxon tests (non‐normal distribution) were performed. Differences in timing of LOM and ROM between FBT‐ and IMT‐treated patient groups were analysed with Mann‐Whitney‐U‐test. Correlations between weight parameters at ROM, LOM and premorbid BW status were calculated with Pearson for normally distributed parameters and with Spearman for not normally distributed parameters. All tests were two‐sided with *α* = 0.05.

## 3 Results

### 3.1 Demographic and Clinical Characteristics

Of the originally 132 patients 9–17 years participating in the parent studies diagnosed with restrictive, atypical or binge‐purge AN, eight participants were ineligible due to male sex (*n* = 7) or receiving masculinising hormone therapy (*n* = 1). Of the remaining 124 females, 25 had no 12FU and were excluded from the analyses, leaving a total of 99 participants treated with either IMT (*n* = 50) or FBT (*n* = 49) who were analysed (Table 1). The 25 patients without 12FU did not differ statistically on any of the assessed baseline demographic and illness variables, but their duration of treatment was significantly shorter (*p* = 0.003; Supporting Information S1: Table S1). At baseline, 48 (48.5%) of the 99 analysed participants were < 15 years old, with an average total age 14.8 ± 1.7 years. Twenty‐six patients (26.3%) were < 1st BMI percentile. Three patients took aripiprazole, which is not known to affect the menstrual cycle, and two patients took olanzapine for < 2 months and switched to aripiprazole (Table 1).

**TABLE 1:** Baseline characteristics of adolescents with 12‐month follow‐up data stratified by those with and those without regular menses at 12‐month follow‐up.

	Total included sample (*N* = 99)	Menses at 12FU (*n* = 45)	No menses at 12FU (*n* = 54)	*p*‐value	Effect size
Age (years), mean ± SD [range]	14.8 ± 1.7 [9.6–17.7]	15.3 ± 1.1 [13.0–17.5]	14.4 ± 1.9 [9.6–17.7]	0.006**	0.27
Height (cm), median (IQR)	164.7 (159.0, 170.0)	165.0 (161.3, 171.8)	163.0 (157.8, 170.0)	0.058	0.44
Weight (kg), median (IQR)	43.0 (39.7, 46.0)	44.0 (42.3, 47.2)	41.0 (36.2, 45.1)	0.001**	0.58
BMI (kg/m^2^), median (IQR)	15.6 (14.8, 16.5)	16.2 (15.4, 16.8)	15.1 (14.6, 16.1)	< 0.001***	0.61
Premorbid weight (kg), mean ± SD [range]	52.7 ± 9.5 [25.0–76.0]^a^	56.2 ± 8.1 [41.0–76.0]	49.6 ± 9.7 [25.0–70.0]^a^	< 0.001***	0.35
Weight suppression (kg), mean ± SD [range]	9.9 ± 5.9 [−4.7–26.6]^a^	11.0 ± 6.2 [0.6–26.6]	9.1 ± 5.5 [−4.7–21.2]^a^	0.109	0.16
%mBMI, median (IQR)	78.9 (74.3, 82.8)	80.0 (75.6, 84.2)	78.1 (74.0, 82.5)	0.088	0.41
BMI percentile, median (IQR)	2.0 (0.0, 6.0)	3.0 (0.5, 7.5)	2.0 (0.0, 6.0)	0.265	0.34
Z‐score, median (IQR)	−2.04 (−2.68, −1.58)	−1.95 (−2.57, −1.51)	−2.13 (−2.91, −1.63)	0.134	0.15
CET, median (IQR)	13.3 (10.8, 17.3)^b^	14.4 (12.0, 16.4)^a^	13.0 (9.9, 17.6)^a^	0.399	0.29
EDEQ global, median (IQR)	3.5 (2.0, 4.7)^a^	4.0 (3.1, 4.8)	3.1 (1.8, 4.6)^a^	0.063	0.43
Subtype, *N* (%)				0.308	0.15
Restrictive AN	85 (86%)	36 (80%)	49 (91%)		
Atypical AN	6 (6%)	4 (9%)	2 (4%)		
Binge‐purge AN	8 (8%)	5 (11%)	3 (6%)		
Psychotropic medication, *n* (%)				0.712	−0.04
No	85 (86%)	38 (84%)	47 (87%)		
Yes	14 (14%)	7 (16%)	7 (13%)		
Number of medications, median (IQR)	0.0 (0.0, 0.0)	0.0 (0.0, 0.0)	0.0 (0.0, 0.0)	0.364	0.09
Medication type, *n* (%)					
SSRI/SNRI	8 (8%)	4 (9%)	4 (7%)	1.000	−0.03
SGA	5 (5%)	3 (7%)	2 (4%)	0.657	−0.07
Tranquiliser	0	0	0	—	—
Other	0	0	0	—	—
Comorbidities, *n* (%)				0.025*	0.23
No	55 (56%)	19 (42%)	35 (65%)		
Yes	44 (44%)	26 (58%)	19 (35%)		
Number of comorbidities, median (IQR)	0.0 (0.0, 1.0)	1.0 (0.0, 1.0)	0.0 (0.0, 1.0)	0.039*	0.207
Comorbidities type, *n* (%)					
Depressive disorders	30 (30%)	16 (36%)	14 (26%)	0.299	−0.10
OCD	14 (14%)	8 (18%)	6 (11%)	0.343	−0.10
Anxiety disorders	14 (14%)	10 (22%)	4 (7%)	0.045*	−0.21
Personality pathology	5 (5%)	2 (4%)	3 (6%)	1.000	0.03
Other	3 (3%)	2 (4%)	1 (2%)	0.589	−0.08
Menstruation, *n* (%)					
Secondary amenorrhoea	66 (67%)	34 (76%)	32 (59%)	0.087	−0.17
No menarche	26 (26%)	6 (13%)	20 (37%)	0.008**	0.03
Regular menses	5 (5%)	3 (7%)	2 (4%)	0.657	0.07
Irregular menses	0	0	0	—	—
Contraceptive use < 3 months	2 (2%)	2 (4%)	0	—	—
Duration of treatment (days), median (IQR)	166 (111, 238)^a^	161 (105, 215)^a^	167 (116, 251)	0.345	0.10
IMT	125 (100, 168)	114 (100, 168)	135 (99, 168)	0.477	0.15
FBT	237 (162, 354)^a^	215 (121, 314)^a^	243 (165, 367)	0.311	0.10
Treatment, *n* (%)				0.912	0.01
IMT	50 (51%)	23 (51%)	27 (50%)		
FBT	49 (49%)	22 (49%)	27 (50%)		

*Note:* Effect sizes are Pearson's r or Phi.

Abbreviations: %mBMI, percent median body mass index based on percentile curves by Kromeyer–Hausschild (Germany); 12FU, 12‐month follow‐up; AN, anorexia nervosa; BMI, body mass index; CET, compulsive exercise test; cm, centimetre; EDEQ, eating disorder examination questionnaire; FBT, family‐based treatment; IMT, inpatient multimodal treatment; IQR, interquartile range; kg, kilogramme; OCD, obsessive‐compulsive disorder; SGA, second‐generation antipsychotics; SNRI, serotonin‐norepinephrine reuptake inhibitor; SSRI, selective serotonin reuptake inhibitor.

^a^ Data from one patient were not available.

^b^ Data from two patients were not available.

^*^ *p* < 0.05.

^**^ *p* < 0.01.

^***^ *p* < 0.001.

### 3.2 Menstrual Development

Of 66 patients with secondary amenorrhoea at baseline (Table 1), 27 (40.9%) had regular menses at 6FU and 35 (53.0%) at 12FU, which was a significant increase over 12 months (*p* < 0.001). Six patients with ROM at 6FU lost regular menses at 12FU accompanied by a median %mBMI loss of −4.5 [IQR = −8.4, −1.8], whereas the 21 patients with regular menses at both 6FU and 12FU showed a median %mBMI gain of 0.2 [IQR = −3.8, 2.3]. Of the six patients, four were treated with FBT and two with IMT. For two of the FBT patients, treatment duration was approximately twice the median duration of the total sample, whereas it was close to the median for the remaining four patients. No further abnormalities in medication, comorbidities, subtype and premorbid weight were observed in these patients.

Of 26 premenarchal patients at baseline, seven (26.9%) had menarche at 12FU and 19 remained premenarchal. Of the patients who remained premenarchal, nine (47.4%) were > 15 years old at 12FU, which is considered primary amenorrhoea.

Three of five patients with regular menses at baseline menstruated continually and two had secondary amenorrhoea at 6FU and 12FU. Two patients were using contraceptives at baseline for < 3 months and showed secondary amenorrhoea at 6FU and regular menses at 12FU.

### 3.3 Menses Group Versus No‐Menses Group

At 12FU, regular menses was present in 45 patients (45.5%). Patients who had no menses at 12FU were lower weight at baseline compared to those who had regular menses at 12FU, but there were no differences in weight suppression between the two groups. Patients with regular menses at 12FU had significantly more baseline psychiatric comorbidities, especially anxiety disorders (Table 1). There was no difference in the baseline %mBMI between patients with and without comorbidities (78.9 vs. 81.0).

Table 2 shows baseline and follow‐up data of patients with versus without regular menses at 12FU. Weight gain measured in %mBMI from baseline to 12FU was significant in both groups (*p* < 0.001). While the age‐and‐sex‐related weight metrics at baseline did not differ significantly between the two groups, at 6FU and 12FU the %mBMI, BMI percentile and z‐scores were higher in the menses group (Table 2).

**TABLE 2:** Comparisons of weight parameters from baseline to 6‐ and 12‐month follow‐up between adolescents with or without regular menses at 12‐month follow‐up.

		Total included sample (*N* = 99)	Menses at 12FU (*n* = 45)	No menses at 12FU (*n* = 54)	*p*‐value	Effect size
Height (cm), median (IQR)	BL	164.7 (159.0, 170.0)	165.0 (161.3, 171.8)	163.0 (157.8, 170.0)	0.058	0.44
	6FU	165.0 (160.0, 170.0)	166.0 (162.0, 171.3)	163.0 (157.0, 170.0)	0.032*	0.47
	12FU	165.6 (160.0, 171.0)	166.0 (162.5, 175.8)	166.0 (158.8, 173.0)	0.077	0.42
	Δ (12FU−BL)	0.0 (0.0, 0.0)	0.0 (0.0, 0.0)	0.0 (0.0, 0.0)	0.168	0.37
Weight (kg), median (IQR)	BL	43.0 (39.7, 46.0)	44.0 (42.3, 47.2)	41.0 (36.2, 45.1)	0.001**	0.58
	6FU	50.2 (46.0, 54.1)	52.1 (47.9, 55.8)	48.4 (42.8, 53.1)	0.002**	0.56
	12FU	50.8 (47.6, 54.7)	53.0 (49.8, 58.0)	48.6 (42.0, 52.0)	< 0.001***	0.64
	Δ (12FU–BL)	8.0 (5.1, 11.1)	8.0 (5.6, 11.1)	7.7 (4.4, 11.4)	0.271	0.33
BMI (kg/m^2^), median (IQR)	BL	15.6 (14.8, 16.5)	16.2 (15.4, 16.8)	15.1 (14.6, 16.1)	< 0.001***	0.61
	6FU	18.2 (17.3, 19.1)	18.6 (17.8, 19.7)	17.8 (17.0, 18.5)	0.002**	0.56
	12FU	18.4 (17.3, 19.3)	19.1 (18.2, 20.0)	17.6 (16.7, 18.8)	< 0.001***	0.64
	Δ (12FU–BL)	2.6 (1.7, 3.8)	2.7 (1.8, 4.0)	2.5 (1.7, 3.8)	0.337	0.31
%mBMI, median (IQR)	BL	78.9 (74.3, 82.8)	80.0 (75.6, 84.2)	78.1 (74.0, 82.5)	0.088	0.41
	6FU	89.6 (85.8, 94.2)	92.4 (86.4, 95.9)	88.1 (85.6, 92.7)	0.041*	0.44
	12FU	90.5 (86.5, 96.5)	91.8 (88.5, 98.2)	89.5 (85.1, 92.5)	0.010*	0.51
	Δ (12FU–BL)	11.4 (6.4, 17.1)	11.6 (8.3, 18.2)	10.9 (5.4, 16.6)	0.336	0.31
BMI percentile, median (IQR)	BL	2.0 (0.0, 6.0)	3.0 (0.5, 7.5)	2.0 (0.0, 6.0)	0.265	0.34
	6FU	19.0 (11.0, 32.0)	25.0 (11.5, 37.5)	16.0 (11.0, 28.0)	0.054	0.45
	12FU	20.0 (12.0, 36.0)	25.0 (15.5, 43.0)	18.0 (7.8, 27.5)	0.014*	0.51
	Δ (12FU–BL)	16.0 (8.0, 30.0)	20.0 (10.0, 35.5)	13.5 (4.8, 24.0)	0.069	0.43
Z‐score, median (IQR)	BL	−2.04 (−2.68, −1.58)	−1.95 (−2.57, −1.51)	−2.13 (−2.91, −1.63)	0.134	0.15
	6FU	−0.8 (−1.31, −0.45)	−0.60 (−1.23, −0.33)	−1.02 (−1.37, −0.55)	0.038*	0.21
	12FU	−0.80 (−1.33, −0.34)	−0.61 (−1.02, −0.06)	−1.01 (−1.70, −0.61)	0.002**	0.31
	Δ (12FU–BL)	1.33 (0.73, 1.91)	1.30 (0.84, 1.96)	1.39 (0.59, 1.84)	0.326	0.10

*Note:* Effect sizes are Pearson's r or Phi. At 6FU data from three patients in the group ‘no menses at 12FU’ were not available.

Abbreviations: Δ, delta; %mBMI, percent median body mass index based on percentile curves by Kromeyer–Hausschild (Germany); 12FU, 12‐month follow‐up; BL, baseline BMI, body mass index; centimetre; IQR, interquartile range; kg, kilogramme.

^*^ *p* < 0.05.

^**^ *p* < 0.01.

^***^ *p* < 0.001.

Compulsive exercise and global eating disorder‐specific psychopathology at baseline, 6FU and 12FU did not differ significantly between the two groups. Eating concern at baseline and weight concern and shape concern at 6FU were significantly higher in the menses group while there were no significant differences at other times or in other subscales (Tables 3).

**TABLE 3:** Comparisons of compulsive exercise and eating disorder psychopathology from baseline to 6‐ and 12‐month follow‐up between adolescents with or without regular menses at 12‐month follow‐up.

		Total included sample (*N* = 98)	Menses at 12FU (*n* = 45)	No menses at 12FU (*n* = 53)	*p*‐value	Effect size
CET, *n* (%)	BL	13.3 (10.8, 17.3)	14.4 (12.0, 16.4)	13.0 (9.9, 17.6)	0.399	0.29
	6FU	10.9 (6.5, 14.4)	10.1 (6.4, 13.8)	11.2 (6.6, 15.1)	0.368	0.31
	12FU	10.0 (5.5, 14.7)	10.9 (5.7, 15.4)	10.0 (5.5, 13.9)	0.697	0.21
	Δ (12FU–BL)	−3.0 (−6.3, 1.3)	−2.8 (−5.7, 0.7)	−3.0 (−6.9, 1.7)	0.849	0.15
EDEQ global, *n* (%)	BL	3.5 (2.0, 4.7)	4.0 (3.1, 4.8)	3.1 (1.8, 4.6)	0.063	0.43
	6FU	1.8 (0.9, 3.4)	2.1 (1.0, 3.8)	1.4 (0.8, 2.7)	0.086	0.42
	12FU	1.8 (0.8, 3.8)	1.9 (1.0, 4.3)	1.7 (0.7, 3.3)	0.106	0.42
	Δ (12FU–BL)	−1.0 (−2.2, −0.2)	−1.0 (−2.3, −0.6)	−1.0 (−2.2, 0.0)	0.676	0.21
EDEQ restraint, *n* (%)	BL	3.4 (1.4, 4.8)	3.6 (2.2, 5.0)	3.2 (1.2, 4.8)	0.326	0.32
	6FU	1.2 (0.3, 2.8)	1.2 (0.6, 3.0)	1.2 (0.2, 2.3)	0.325	0.32
	12FU	1.2 (0.2, 3.7)	1.1 (0.2, 4.4)	1.2 (0.3, 2.9)	0.524	0.26
	Δ (12FU–BL)	−1.0 (−2.9, 0.0)	−1.0 (−3.0, 0.1)	−0.8 (−2.8, 0.0)	0.811	0.16
EDEQ eating concern, *n* (%)	BL	2.8 (1.8, 4.0)	3.4 (2.2, 4.3)	2.6 (1.6, 3.6)	0.038*	0.46
	6FU	1.2 (0.4, 2.4)	1.4 (0.6, 2.8)	1.1 (0.4, 2.2)	0.211	0.36
	12FU	1.2 (0.4, 2.6)	1.4 (0.4, 3.3)	1.1 (0.4, 2.2)	0.276	0.34
	Δ (12FU–BL)	−1.0 (−1.9, −0.2)	−1.1 (−2.0, −0.2)	−0.8 (−1.8, 0.0)	0.355	0.32
EDEQ weight concern, *n* (%)	BL	4.2 (1.4, 5.3)	3.6 (2.7, 5.2)	2.6 (1.4, 5.0)	0.116	0.40
	6FU	1.8 (0.8, 3.7)	2.4 (1.0, 4.0)	1.3 (0.6, 2.8)	0.032*	0.47
	12FU	2.0 (0.6, 4.1)	2.4 (1.0, 4.6)	1.8 (0.5, 3.6)	0.221	0.36
	Δ (12FU–BL)	−0.8 (−2.1, 0.1)	−1.1 (−2.1, 0.0)	−0.6 (−2.2, 0.2)	0.434	0.29
EDEQ shape concern, *n* (%)	BL	4.4 (2.8, 5.6)	4.9 (3.6, 5.3)	4.1 (2.5, 5.5)	0.198	0.36
	6FU	2.9 (1.5, 4.8)	3.4 (1.8, 5.3)	2.3 (1.0, 4.3)	0.040*	0.46
	12FU	2.4 (1.1, 5.0)	2.8 (1.4, 5.2)	2.4 (0.9, 4.7)	0.177	0.38
	Δ (12FU–BL)	−0.8 (−2.6, 0.0)	−0.8 (−3.0, 0.0)	−0.6 (−2.5, 0.0)	0.545	0.26

*Note:* Effect sizes are Pearson's r or Phi. At baseline CET data from one patient in the group ‘menses at 12FU’ were not available. At 6FU CET data from seven patients (42 vs. 49) and EDEQ data from five patients (43 vs. 50) were not available. At 12FU CET data from 15 patients (37 vs. 46) and EDEQ data from 12 patients (38 vs. 48) were not available. The difference in CET Δ (12FU–BL) was calculated for 36 patients in the group ‘menses at 12FU’ and for 45 patients in the group ‘no menses at 12FU’. The difference in EDEQ Δ (12FU–BL) was calculated for 38 patients in the group ‘menses at 12FU’ and for 47 patients in the group ‘no menses at 12FU’.

Abbreviations: Δ, delta; 12FU, 12‐month follow‐up; BL, baseline; CET, compulsive exercise test; EDEQ, eating disorder examination questionnaire; kg, kilogramme.

^*^ *p* < 0.05.

[Correction added on 21 August 2026, after first online publication: The sample size in the column heading “Menses at 12FU (*n* = 44)” has been corrected to (*n* = 45). In addition, the text “Cox & Snell *R* ^2^ = 0.188. Nagelkerke's *R* ^2^ = 0.251. Area under the curve (AUC) = 0.753.” has been moved from Table 3 note to Table 4 note.]

### 3.4 Predictors for Regular Menses at 12‐Month Follow‐Up

Baseline and 12FU parameters with significant differences between patients with versus without regular menses at 12FU (see Tables 1 and 2) were included into a multivariable stepwise backward logistic regression model to predict regular menses at 12FU. The most significant age‐and‐sex‐adjusted weight metric was selected among all collinear weight parameters. The following baseline parameters were included in the multivariable regression model: age, %mBMI, presence of psychiatric comorbidities (yes/no), menarche, and EDEQ Eating Concern; 12FU parameter: %mBMI.

The final multivariable logistic regression model (Table 4) showed that in the total sample presence of menses at 12FU was significantly and independently related to presence of menarche at baseline (OR = 6.036, 95%CI = 1.915–19.029) and higher %mBMI at 12FU (OR = 1.084, 95%CI = 1.024–1.149), Nagelkerke's (*R* ^2^ = 0.251).

**TABLE 4:** Multivariable stepwise backward logistic regression model for menses at 12‐month follow‐up. *N* = 98.

Independent predictors for menses at 12FU	Regression coefficient B	SE	Odds ratio	*p*‐value	95% CI
Presence of menarche at BL	1.798	0.586	6.036	0.002*	1.915–19.029
12FU %mBMI	0.081	0.029	1.084	0.006*	1.024–1.149

*Note:* Cox & Snell *R* ^2^ = 0.188. Nagelkerke's *R* ^2^ = 0.251. Area under the curve (AUC) = 0.753.

Abbreviations: %mBMI, percentage of the median body mass index based on percentile curves by Kromeyer–Hausschild (Germany); 12FU, 12‐month follow‐up; B, unstandardised regression coefficient; BL, baseline; CI, confidence interval; SE, standard error.

^*^ *p* < 0.05.

To eliminate possible bias due to premenarchal patients, the univariate analyses were also performed in a subgroup, with 66 patients who had secondary amenorrhoea at baseline (35 with vs. 31 without regular menses at 12FU; Supporting Information S1: Tables S2–S4). Parameters with significant differences between groups were included in the multivariable stepwise backward logistic regression model, that is, %mBMI at baseline and %mBMI at 12FU. The final multivariable model retained both variables and showed that presence of menses at 12FU in girls with secondary amenorrhoea was significantly and independently related to a higher %mBMI at baseline (OR = 1.137, 95%CI = 1.022–1.264) and %mBMI at 12FU (OR = 1.102, 95%CI = 1.018–1.192; Nagelkerkes *R* ^2^ = 0.308) (Supporting Information S1: Table S5).

When looking at patients without menarche at baseline (5 with vs. 21 without regular menses at 12FU; Supporting Information S1: Tables S6–S8) age‐and‐sex‐adjusted weight parameters did not differ significantly between groups. Compulsive exercise at 6FU (*p* = 0.009) and global eating disorder‐specific psychopathology and 6FU (*p* = 0.009) and at 12FU (*p* = 0.004) were higher in the menses group but due to the small number of cases, no regression analysis was carried out.

### 3.5 Weight and Timing of Loss and Resumption of Menses

Forty‐one patients, representing 50% of those with secondary amenorrhoea at baseline, provided information on weight, height and time of LOM and ROM (Table 5). The frequency of atypical AN, binge purging, and depressive disorders at baseline was significantly higher in this sample compared to the 41 patients with secondary amenorrhoea who did not provide detailed information on LOM and ROM (Supporting Information S1: Table S1).

**TABLE 5:** Premorbid weight parameters and parameters at resumption of menses and loss of menses in adolescents who completed the additional menstrual questionnaire.

	Premorbid (*n* = 41)	LOM (*n* = 41)	ROM (*n* = 41)	Premorbid versus ROM		LOM versus ROM	
				*p*‐value	Correlation (*p*‐value)	*p*‐value	Correlation (*p*‐value)
Age (years), median (IQR) [range]	13.7 (12.9, 15.0) [10.5–16.2]	14.1 (13.4, 15.3) [12.3–16.9]	15.3 (14.7, 17.3) [13.6–18.9]	< 0.001***	0.78 (< 0.001***)	< 0.001***	0.73 (< 0.001***)
Height (cm), median (IQR) [range]	165.0 (160.0, 170.0) [153.0–181.0]	165.0 (160.0, 171.0) [155.0–181.0]	165.0 (162.5, 172.3) [156.0–181.0]	< 0.001***	0.96 (< 0.001***)	< 0.001***	0.97 (< 0.001***)
Weight (kg), median (IQR) [range]	53.0 (48.0, 59.0) [41.0–70.0]	47.5 (42.4, 50.0) [28.0–65.0]	53.0 (48.0, 56.3) [41.0–65.0]	0.179	0.66 (< 0.001***)	< 0.001***	0.79 (< 0.001***)
BMI (kg/m^2^), median (IQR) [range]	19.5 (18.0, 20.9) [15.9–27.3]	17.1 (16.0, 18.3) [10.9–22.0]	18.6 (17.6, 19.9) [15.4–21.8]	0.013*	0.58 (< 0.001***)	< 0.001***	0.57 (< 0.001***)
%mBMI, median (IQR) [range]	98.5 (94.1, 109.7) [80.1–133.8]	85.2 (79.9, 92.0) [31.1–109.5]	92.0 (86.6, 97.8) [76.6–103.4]	< 0.001***	0.58 (< 0.001***)	0.001**	0.47 (0.002**)
BMI percentile, median (IQR) [range]	45.0 (31.5, 73.0) [3.0–97.0]	13.0 (5.0, 26.5) [0.0–74.0]	25.0 (12.0, 42.5) [1.0–60.0]	< 0.001***	0.56 (< 0.001***)	0.006**	0.50 (0.001**)
Z‐score, median (IQR) [range]	−0.11 (−0.48, 0.62) [−1.87–1.85]	−1.13 (−1.69, −0.61) [−5.57–0.62]	−0.62 (−1.11, −0.16) [−2.39–0.39]	< 0.001***	0.44 (0.001**)	0.001**	0.41 (0.008**)

*Note:* Correlation was calculated with Pearson for normally distributed parameters and with Spearman for non‐normally distributed parameters.

Abbreviations: %mBMI, percent median body mass index based on percentile curves by Kromeyer–Hausschild (Germany); BMI, body mass index; cm, centimetre; IQR, interquartile range; kg, kilogramme; LOM, loss of menses; ROM, resumption of menses; versus, versus.

^*^ *p* < 0.05.

^**^ *p* < 0.01.

^***^ *p* < 0.001.

Table 5 shows age, height and weight parameters at premorbid status, LOM and ROM in the subgroup of 41 patients. The median BMI percentile at ROM was 25 (range = 1.0–60.0) equaling the %mBMI of 92.0 [IQR = 86.6, 97.8], which was significantly higher than the %mBMI at LOM of 85.2 [IQR = 79.9, 92.0] (*p* = 0.001) but significantly lower than the premorbid %mBMI 98.5 [IQR = 94.1, 109.7] (*p* < 0.001).

The median duration from illness onset to LOM was 2.5 months [IQR = 1.0, 6.3], the median duration of amenorrhoea was 15 months [IQR = 12.0, 25,0] and the median duration from baseline to ROM was 8 months [IQR = 5.0, 15.5]. The timings were similar for FBT‐ and IMT‐treated patients (*p* = 0.405; *p* = 0.742; *p* = 0.979, respectively).

### 3.6 Comparison of Family‐Based Treatment and Inpatient Multimodal Treatment Regarding Menstrual Development

There were no significant baseline differences between patients receiving IMT or FBT. The percentage of patients with regular menses at 12FU (Table 1) was similar in FBT‐ and IMT‐treated patients (*p* = 0.912). Likewise, FBT‐ and IMT‐treated patients did not differ significantly regarding the median duration from illness onset to LOM (*p* = 0.405), median duration of amenorrhoea (*p* = 0.742), and median duration from baseline to ROM (*p* = 0.979).

## 4 Discussion

This study examined menstrual trajectories over 12 months in female adolescents with AN who received either IMT or FBT. Sixty‐seven percent of patients had secondary amenorrhoea at baseline, and 53% of these showed ROM at 12FU. Although these rates were lower than hypothesised, they were consistent in directionality with our expectations. Predictors for achieving regular menses at 12FU were menarche at baseline and a higher %mBMI at 12FU, as hypothesised, while baseline eating disorder‐specific psychopathology, contrary to our hypothesis, was not associated with menses at 12FU. The median BMI percentile at ROM was 25, range 1.0–60.0 (%mBMI = 92.0), which was correlated with the mean BMI percentile at LOM and with the premorbid mean BMI percentile, as hypothesised. Contrary to our hypothesis, there was no difference in the frequency or timing of ROM in FBT versus IMT patients, although ROM was a defined treatment goal in FBT.

### 4.1 Course of Menstrual Status Over Time

No patients were excluded from our study because of their menstrual status, in contrast most previous studies in adolescents and young adults with AN excluded premenarchal patients (Faust et al. 2013; Golden et al. 2008; Pitts et al. 2014). This fact allowed for a comprehensive depiction of the distribution of menstrual status at baseline. Studies by Dempfle et al. 2013 and Pruccoli et al. 2023 also included premenarchal patients, reporting that at baseline, one‐fifth of adolescent patients had not reached menarche, which is congruent with a quarter of patients in our sample.

Dempfle et al. 2013 also showed that 47% of participants were menstruating regularly at 12FU, consistent with the 45% observed in our sample. In the subgroup of patients with secondary amenorrhoea, our study found 53% menstruating at 12FU. This is slightly lower than that reported in other studies, where ∼67% of adolescents (Golden et al. 2008; Faust et al. 2013) reported ROM at 12FU and similar rates were observed in young adults within 5 years (Rastogi et al. 2020). This discrepancy may be attributable to the definition of regular menses being after a single menstrual cycle (Faust et al. 2013; Rastogi et al. 2020), compared to the more conservative definition of ≥ 3 consecutive cycles in our study and in Golden et al. 2008.

### 4.2 Predictors for Regular Menses at 12‐Month Follow‐Up

In this study, the presence of menarche at baseline and the %mBMI at 12FU were the most relevant associated factors for regular menses at 12FU. Patients with menarche at baseline had a 6‐fold higher chance of menstruating regularly at 12FU compared to premenarchal patients, aligning with Dempfle et al. 2013, who reported an even higher odds of a 14‐fold increased chance.

Consistent with previous studies, weight parameters at 12FU of patients menstruating at 12FU were significantly higher than in patients not menstruating, despite a clinically important wide overlap in weight parameters between groups (Golden et al. 2008; Faust et al. 2013; Dempfle et al. 2013). While this difference was only numerical without reaching statistical significance in two studies (Golden et al. 2008; Faust et al. 2013), the difference was statistically significant in Dempfle et al. 2013 where the %mBMI at 12FU was strongly correlated with ROM, as in our study. Additionally, Dempfle et al. 2013 found a longer duration of hospital treatment in inpatients and day‐hospital patients as a prognostic factor for continued amenorrhoea, while in our study no difference in treatment duration between menses and no‐menses group was found even when considering only IMT patients.

When looking separately at patients with secondary amenorrhoea at baseline, our study found that ROM was associated with higher weight parameters at both baseline and 12FU, but not with other parameters, including eating disorder‐specific psychopathology. In contrast, Faust et al. 2013 examined adolescent FBT patients, reporting a positive association between high baseline eating disorder‐specific psychopathology and ROM, but no association between ROM and baseline %expected BW. This finding is contrary to a study in adults that reported a negative association between baseline eating disorder‐specific psychopathology and ROM (Castellini et al. 2020). Further studies are needed to understand how adolescent eating disorder‐specific psychopathology is related to menstrual recovery in different treatment settings and study samples.

So far, predictors for the onset of menses in premenarchal patients with AN have not been investigated. In our study, premenarchal patients at baseline who reported regular menses at 12FU had similar age‐and‐sex‐adjusted weight parameters compared to continuing premenarchal patients at all time‐points. However, contrary to our expectations they had significantly higher compulsive exercise and eating disorder‐specific psychopathology at 6FU and 12FU. Onset of menses may encourage behaviour that counteracts weight gain. However, it could also represent a subgroup with particularly pronounced thoughts related to eating disorders and compulsive exercise. For example, Pruccoli et al. 2023 characterised patients with a premenarchal AN diagnosis as a specific subgroup of AN with unique endocrine and prognostic features that should be studied in more detail.

### 4.3 Correlations Between Premorbid Weight Status, Loss of Menses and Resumption of Menses

This study found that median BMI percentile at ROM was 25 (%mBMI = 92.0) which precisely aligns with the current recommendations of the German S3‐guidelines (Herpertz et al. 2019), confirming the body of evidence on which these guidelines are based (Golden et al. 2008; Swenne 2004; Dempfle et al. 2013). However, we also found that 70% of patients had higher (> 30th) or lower (< 20th) BMI percentiles at ROM. This extends the findings of Swenne 2004, which showed that ROM occurs across a wide range of 30 kg for weight and 6 kg/m^2^ for BMI. These findings suggest that additional studies are needed to investigate parameters beyond anthropometric, AN and other psychiatric illness parameters, or psychophysiological and behavioural variables that may be more closely related to LOM and ROM. These could include detailed assessments of fat mass and distribution, sex hormone levels, leptin levels, inflammatory markers, and other endocrine parameters, including prolactin levels that can be increased during times of stress and also reduce the likelihood of ROM (Dei et al. 2008; Cominato et al. 2014; El Ghoch et al. 2016).

In our study, premorbid age‐and‐sex‐adjusted weight parameters were higher than those at ROM in 78.0% of patients. This finding indicates that full restoration of premorbid age‐and‐sex‐adjusted weight is not a prerequisite for the first sign of physiological recovery manifesting as menstruation. Similarly, previous studies in adolescents (Swenne 2004), and in both adolescents and adults (Favaro and Santonastaso 2009), reported that the absolute weight at ROM was approximately 2 kg below the premorbid weight in most patients. In comparison with a previous study of patients aged 13–34 years at the onset of amenorrhoea (Favaro and Santonastaso 2009), our results show higher rates of ROM at weights exceeding premorbid weight by > 2 kg (19.5% vs. 8%) and within ± 2 kg of premorbid weight (36.6% vs. 24%), and lower rates at weights > 2 kg below premorbid weight (43.9% vs. 68%). However, we included more younger patients whose weight changed considerably during growth, leading to higher absolute weight differences between ROM and premorbid weight. As was the case in our study, Favaro and Santonastaso 2009 used the weight just before illness onset as their premorbid reference, rather than using the maximum premorbid weight, as other studies had done (Swenne 2004; Rastogi et al. 2020). Nevertheless, when studying adolescents, who are still developing, both definitions are likely quite similar (Favaro and Santonastaso 2009) because the growth hormone axis is usually suppressed before the gonadal axis during weight loss (Hill and Elias 2018).

In our study, patients reported LOM after a median weight loss of 7.8 kg. Similar findings were reported by Swenne 2004, who observed a mean weight loss of 7.3 kg. Swenne 2004 also reported a median duration of amenorrhoea of 9 months, which is considerably shorter than the 15 months duration observed in our study. In the cohort by Swenne (2004), fewer than half of the patients had amenorrhoea lasting > 1 year, whereas this applied to 63.4% of our sample. The authors emphasised that the duration of amenorrhoea varied considerably (Swenne 2004). Differences in findings may be attributable to differences in study populations, as patients with eating disorder not otherwise specified of the restrictive subtype were included in the study by Swenne 2004.

The higher weight at ROM than at LOM may reflect that reproductive capacity is only restored once the body has corrected the energy deficit and built up reserves in the form of adipose tissue (Pitts et al. 2014). Additionally, hormonal normalisation seems to take longer than weight recovery. The release of gonadotropin‐releasing hormone often remains suppressed even when weight increases, as leptin levels must reach a certain level to reactivate hypothalamic function. Even after weight recovery, psychological stress, excessive exercise or restrictive eating behaviour can hamper cortisol normalisation which influences the reproductive endocrine axis (Haines 2023). In summary, higher weight parameters are generally required to achieve ROM than those present at LOM. However, full premorbid BMI percentile restoration does not appear to be required for ROM in most patients.

### 4.4 Family‐Based Treatment Versus Inpatient Multimodal Treatment

Contrary to our hypothesis, our study found no difference between patients treated with FBT and those who received IMT regarding the prevalence of menses at 12FU, duration of amenorrhoea, or the time to ROM from baseline. To our knowledge, no previous study has compared menstrual recovery between patients undergoing FBT versus IMT.

### 4.5 Limitations and Clinical Relevance

While this study has several strengths, including the consideration of participants regardless of their menstrual status, a broad examination of this status across multiple dimensions, and being the first study to directly compare ROM between FBT‐ and IMT‐treated adolescent groups, it also has several limitations.

First, the sample size for analyses comparing ROM to LOM and premorbid parameters was relatively small, which may limit statistical power to find significant group differences. However, there were no clinically relevant differences in baseline parameters between patients who provided this information and those who did not, indicating that the subgroup may accurately represent the entire group. Second, no hormonal assessments, pelvic ultrasounds, or measures of body fat were included, which restricts the physiological interpretation of menstrual status. Third, data on premorbid weight and height were collected retrospectively being vulnerable to recall bias. Fourth, all data on menses were self‐reported. Adolescents with AN may underreport or inaccurately recall their menstrual history, leading to potential misclassification. Fifth, irregular menstrual cycles are common in healthy adolescent girls, especially within the first 2 years post‐menarche (Harley et al. 2024; American College of Obstetricians and Gynecologists 2015), which may complicate the interpretation of menstrual (ir)regularity in this population. Finally, although the FBT and IMT groups were collected in the same clinical setting, with very similar in‐ and exclusion criteria, and at largely overlapping times, patients were derived from separate studies.

Despite these limitations, our study serves to better delineate the course and predictors of menstrual function in adolescent females with AN, which is a central part of recovery, as ROM not only indicates the restoration of reproductive health, but also points to the normalisation of hormonal functions that are crucial for bone health (Gibson et al. 2024; Misra et al. 2008) and brain development (Mainz et al. 2012), and that may affect neurocognition, anxiety, depression and eating disorder‐specific psychopathology (Misra and Klibanski 2014).

### 4.6 Conclusions

In conclusion, this study highlights the complex and heterogeneous course of menstrual recovery in adolescent females with AN. Nearly half of the participants resumed regular menses at 12FU. ROM was predicted by the presence of menarche at baseline and a higher %mBMI at 12FU. These findings emphasise the importance of focussing on preventing massive weight loss prior to entering treatment and on early and sufficient weight restoration when working towards menstrual recovery. This focus does not only apply to defining individual target weights, but also to making appropriate adjustments of the weight target range during treatment. Although ROM occurred at a median of the 25th BMI percentile, this threshold does not represent an appropriate target for all patients, as there was considerable variation within a wide BMI percentile range. Age‐and‐sex‐adjusted weight parameters at premorbid status and at LOM can help predict the weight and timing of ROM and should be considered when setting an individual healthy weight target range. It is important to note that not all patients need to reach their premorbid BMI percentile to achieve ROM. Further studies are needed to explore the mechanisms and predictors of reproductive recovery, particularly in premenarchal adolescents with AN, whose menstrual trajectory remains poorly understood. Additionally, while weight gain appears critical for ROM, the relationship between physical and psychological recovery remains unclear. It is probable that improvements in psychological health lag behind physical recovery, as reflected by the similar eating disorder‐specific psychopathology in those who had and who had not restored menses at 12FU. Furthermore, future studies on recovery from amenorrhoea in AN should assess body composition and hormone levels prospectively from admission to discharge and extended follow‐up to generate a comprehensive biological model of ROM in adolescents and to develop more individualised treatment strategies.

## Author Contributions

**Annika Woltmann:** methodology, investigation, data management, writing – original draft. **Linus Imken:** writing – review and editing. **Vivien Kaiser:** investigation, data management. **Johanna L. Keeler:** writing – review and editing. **Daniel Le Grange:** writing – review and editing. **Christoph U. Correll:** conceptualization, methodology, investigation, data management, writing – review and editing, supervision, funding acquisition. **Verena Haas:** conceptualization, methodology, writing – review and editing, project administration, funding acquisition. All authors have read and agreed to the final version of the submitted version of the manuscript.

## Funding

Partial funding for this study was provided by the Christina‐Barz‐Foundation (grant number: T0155‐31.954, PI: V Haas) and Elsa‐Neumann‐Scholarship in accordance with the Berlin State Act to Support Early Career Researchers and Artists (Nachwuchsförderungsgesetz, NaFöG), Humboldt Universität zu Berlin, Humboldt Graduate School, Unter den Linden 6, 10,099 Berlin, Germany (grant number: H67029, PI V Haas).

## Ethics Statement

Data were used from two consecutive inpatient multimodal treatment (IMT) studies and two consecutive family‐based treatment (FBT) studies. Charité‐Universitätsmedizin Berlin ethics committee approved these studies (reference number IMT: ES2/051/18, EA2/240/22; FBT: EA2/133/19, EA2/063/23). All legal guardians provided written informed consent and participants provided written assent before participating in either study, which were all conducted in accordance with the Declaration of Helsinki on ‘Ethical Principles for Medical Research Involving Human Subjects’.

## Conflicts of Interest

The authors declare no conflicts of interest. CU Correll declares the following interest outside of the content of the present article: consultant and/or advisor to or receipt of honoraria from: AbbVie, Alkermes, Allergan, Angelini, Aristo, Autobahn, Axsome, Boehringer‐Ingelheim, Bristol‐Meyers Squibb, Cardio Diagnostics, Casasco, Cerevel, CNX Therapeutics, Compass Pathways, Darnitsa, Delpor, Denovo, Draig, Eli Lilly, Eumentis Therapeutics, Gedeon Richter, GH Research, Hetero, Hikma, Holmusk, IntraCellular Therapies, Jamjoom Pharma, Janssen/J&J, Karuna, LB Pharma, Lundbeck, MedInCell, MedLink Global, Merck, Mindpax, Mitsubishi Tanabe Pharmaceuticals, Maplight, Mylan, Neumora Therapeutics, Neuraxpharm, Neurocrine, Neurelis, Neurosterix, NeuShen, Neusignal Therapeutics, Newron, Noven, Novo Nordisk, Orion Pharma, Otsuka, PPD Biotech, Recognify Life Science, Recordati, Relmada, Response Pharmaeutical, Reviva, Roemmers, Rovi, Saladax, Sanofi, Seqirus, Servier, Sumitomo Pharma America, Sunovion, Sun Pharma, Supernus, Tabuk, Takeda, Teva, Terran, Tolmar, Vertex, Viatris and Xenon Pharmaceuticals. Expert testimony for Janssen, Lundbeck, Neurocrine, and Otsuka. Data Safety Monitoring Board member/chair for Compass Pathways, IntraCellular Therapies, Relmada, Reviva, Rovi. Research grant support to the institution from Boehringer‐Ingelheim, Janssen and Takeda. Royalties from UpToDate and stock option or stock holder of Cardio Diagnostics, Kuleon Biosciences, LB Pharmaceuticals, MedLink Global, Mindpax, Quantic, Terran.

## Supporting information


Supporting Information S1

## Data Availability

Research data are not shared due to privacy restrictions.

## References

[erv70139-bib-0001] American College of Obstetricians and Gynecologists . 2015. “ACOG Committee Opinion No. 651: Menstruation in Girls and Adolescents: Using the Menstrual Cycle as a Vital Sign.” Obstetrics & Gynecology 126: e143–e146. 10.1097/AOG.0000000000001215. 26595586

[erv70139-bib-0002] Association, A. P. 2022. Diagnostic and Statistical Manual of Mental Disorders. American Psychiatric Association Publishing.

[erv70139-bib-0003] Beumont, P. , P. Hay , D. Beumont , et al. 2004. “Australian and New Zealand Clinical Practice Guidelines for the Treatment of Anorexia Nervosa.” Australian and New Zealand Journal of Psychiatry 38, no. 9: 659–670. 10.1080/j.1440-1614.2004.01449.x. 15324328

[erv70139-bib-0004] Brix, N. , A. Ernst , L. L. B. Lauridsen , et al. 2019. “Timing of Puberty in Boys and Girls: A Population‐Based Study.” Paediatric & Perinatal Epidemiology 33, no. 1: 70–78. 10.1111/ppe.12507. PMC6378593 30307620

[erv70139-bib-0005] Castellini, G. , E. Rossi , E. Cassioli , et al. 2020. “Predictors of Resumption of Menses in Anorexia Nervosa: A 4‐Year Longitudinal Study.” Psychosomatic Medicine 82, no. 8: 782–786. 10.1097/psy.0000000000000849. 32796334

[erv70139-bib-0006] Cominato, L. , M. M. X. Da Silva , L. Steinmetz , V. Pinzon , B. Fleitlich‐Bilyk , and D. Damiani . 2014. “Menstrual Cycle Recovery in Patients With Anorexia Nervosa: The Importance of Insulin‐Like Growth Factor 1.” Hormone Research in Paediatrícs 82, no. 5: 319–323. 10.1159/000367895. 25322903

[erv70139-bib-0007] Couturier, J. , L. Isserlin , M. Norris , et al. 2020. “Canadian Practice Guidelines for the Treatment of Children and Adolescents With Eating Disorders.” Journal of Eating Disorders 8, no. 1: 4. 10.1186/s40337-020-0277-8. PMC6995106 32021688

[erv70139-bib-0008] Crone, C. , L. J. Fochtmann , E. Attia , et al. 2023. “The American Psychiatric Association Practice Guideline for the Treatment of Patients With Eating Disorders.” Australian Journal of Pharmacy 180, no. 2: 167–171. 10.1176/appi.ajp.23180001. 36722117

[erv70139-bib-0009] Dei, M. , V. Seravalli , V. Bruni , D. Balzi , and A. Pasqua . 2008. “Predictors of Recovery of Ovarian Function After Weight Gain in Subjects With Amenorrhea Related to Restrictive Eating Disorders.” Gynecological Endocrinology 24, no. 8: 459–464. 10.1080/09513590802246141. 18850384

[erv70139-bib-0010] Dempfle, A. , B. Herpertz‐Dahlmann , N. Timmesfeld , et al. 2013. “Predictors of the Resumption of Menses in Adolescent Anorexia Nervosa.” BMC Psychiatry 13, no. 1: 308. 10.1186/1471-244x-13-308. PMC3832684 24238469

[erv70139-bib-0011] El Ghoch, M. , S. Calugi , E. Chignola , P. V. Bazzani , and R. Dalle Grave . 2016. “Body Fat and Menstrual Resumption in Adult Females With Anorexia Nervosa: A 1‐Year Longitudinal Study.” Journal of Human Nutrition and Dietetics 29, no. 5: 662–666. 10.1111/jhn.12373. 27230963

[erv70139-bib-0012] Engelhardt, C. , M. Föcker , K. Bühren , et al. 2021. “Age Dependency of Body Mass Index Distribution in Childhood and Adolescent Inpatients With Anorexia Nervosa With a Focus on DSM‐5 and ICD‐11 Weight Criteria and Severity Specifiers.” European Child & Adolescent Psychiatry 30, no. 7: 1081–1094. 10.1007/s00787-020-01595-4. PMC8295075 32666204

[erv70139-bib-0013] Faust, J. P. , A. B. Goldschmidt , K. E. Anderson , et al. 2013. “Resumption of Menses in Anorexia Nervosa During a Course of Family‐Based Treatment.” Journal of Eating Disorders 1: 12. 10.1186/2050-2974-1-12. PMC4053648 24926411

[erv70139-bib-0014] Favaro, A. , and P. Santonastaso . 2009. “Seasonality and the Prediction of Weight at Resumption of Menses in Anorexia Nervosa.” Fertility and Sterility 91, no. 4: 1395–1397. 10.1016/j.fertnstert.2008.04.055. 18692820

[erv70139-bib-0015] Galmiche, M. , P. Déchelotte , G. Lambert , and M. P. Tavolacci . 2019. “Prevalence of Eating Disorders Over the 2000–2018 Period: A Systematic Literature Review.” American Journal of Clinical Nutrition 109, no. 5: 1402–1413. 10.1093/ajcn/nqy342. 31051507

[erv70139-bib-0016] Gasner, A. , and A. Rehman . 2025. Primary Amenorrhea. StatPearls. 32119356

[erv70139-bib-0017] Gibson, D. , Z. Filan , P. Westmoreland , and P. S. Mehler . 2024. “Loss of Bone Density in Patients With Anorexia Nervosa Food That Alone Will Not Cure.” Nutrients 16, no. 21: 3593. 10.3390/nu16213593. PMC11547391 39519426

[erv70139-bib-0018] Golden, N. H. , M. S. Jacobson , W. M. Sterling , and S. Hertz . 2008. “Treatment Goal Weight in Adolescents With Anorexia Nervosa: Use of BMI Percentiles.” International Journal of Eating Disorders 41, no. 4: 301–306. 10.1002/eat.20503. 18176951

[erv70139-bib-0019] Golden, N. H. , D. K. Katzman , R. E. Kreipe , et al. 2003. “Eating Disorders in Adolescents: Position Paper of the Society for Adolescent Medicine.” Journal of Adolescent Health 33, no. 6: 496–503. 10.1016/s1054-139x(03)00326-4. 14642712

[erv70139-bib-0020] Gradl‐Dietsch, G. , T. Peters , A. Meule , J. Hebebrand , and U. Voderholzer . 2024. “Body Mass Index Distribution in Female Child, Adolescent and Adult Inpatients With Anorexia Nervosa‐A Retrospective Chart Review.” Nutrients 16, no. 11: 1732. 10.3390/nu16111732. PMC11175141 38892665

[erv70139-bib-0021] Gräfe, D. n.d. “Ped(z) Kinderarztrechner.” Accessed September 30, 2023. https://www.pedz.de/de/willkommen.html.

[erv70139-bib-0022] Haas, V. , K. Wechsung , V. Kaiser , et al. 2024. “Comparing Family‐Based Treatment With Inpatient Treatment in Youth With Anorexia Nervosa Eligible for Hospitalization: A 12‐Month Feasibility Study.” International Journal of Eating Disorders 57, no. 2: 388–399. 10.1002/eat.24098. 38082440

[erv70139-bib-0023] Haines, M. S. 2023. “Endocrine Complications of Anorexia Nervosa.” Journal of Eating Disorders 11, no. 1: 24. 10.1186/s40337-023-00744-9. PMC9933399 36793059

[erv70139-bib-0024] Harley, K. G. , A. Watson , S. Robertson , V. J. Vitzthum , and A. Shea . 2024. “Menstrual Cycle Characteristics of U. S. Adolescents According to Gynecologic Age and Age at Menarche.” Journal of Pediatric and Adolescent Gynecology 37, no. 4: 419–425. 10.1016/j.jpag.2024.03.005. 38570085

[erv70139-bib-0025] Herpertz, S. , M. Fichter , B. Herpertz‐Dahlmann , et al. 2019. S3‐Leitlinie Diagnostik und Behandlung der Essstörungen. Springer Berlin Heidelberg.

[erv70139-bib-0026] Herpertz‐Dahlmann, B. 2021. “Intensive Treatments in Adolescent Anorexia Nervosa.” Nutrients 13, no. 4: 1265. 10.3390/nu13041265. PMC8068891 33924294

[erv70139-bib-0027] Hilbert, A. , B. Tuschen‐Caffier , A. Karwautz , H. Niederhofer , and S. Munsch . 2007. “Eating Disorder Examination‐Questionnaire.” Diagnostica 53: 144–154. 10.1026/0012-1924.53.3.144.

[erv70139-bib-0028] Hill, J. W. , and C. F. Elias . 2018. “Neuroanatomical Framework of the Metabolic Control of Reproduction.” Physiological Reviews 98, no. 4: 2349–2380. 10.1152/physrev.00033.2017. PMC6170978 30109817

[erv70139-bib-0029] International Statistical Classification of Diseases and Related Health Problems, Geneva, 2004.

[erv70139-bib-0030] Jary Franklin, J. M. , S. L. Winnie , N. Prohaska , T. Bravender , and J. L. van Huysse . 2024. “Estimating Treatment Goal Weights in Adolescents With Anorexia Nervosa and Atypical Anorexia Nervosa: Comparison of the Median BMI and Historical BMI Percentile.” International Journal of Eating Disorders 57, no. 12: 2491–2496. 10.1002/eat.24298. PMC11629056 39324372

[erv70139-bib-0031] Karountzos, V. , I. Lambrinoudaki , A. Tsitsika , and E. Deligeoroglou . 2017. “The Role of Total Body Fat Mass and Trunk Fat Mass, Combined With Other Endocrine Factors, in Menstrual Recovery and Psychopathology of Adolescents With Anorexia Nervosa.” Gynecological Endocrinology 33, no. 10: 757–762. 10.1080/09513590.2017.1334199. 28562123

[erv70139-bib-0032] Kaufman, J. , B. Birmaher , D. Brent , et al. 1997. “Schedule for Affective Disorders and Schizophrenia for School‐Age Children‐Present and Lifetime Version (K‐SADS‐PL): Initial Reliability and Validity Data.” Journal of the American Academy of Child & Adolescent Psychiatry 36, no. 7: 980–988. 10.1097/00004583-199707000-00021. 9204677

[erv70139-bib-0033] Kromeyer‐Hauschild, K. , M. Wabitsch , D. Kunze , et al. 2001. “Perzentile für den Body‐Mass‐Index für das Kindes‐ und Jugendalter unter Heranziehung verschiedener deutscher Stichproben.” Monatsschrift Kinderheilkunde.

[erv70139-bib-0034] Legroux‐Gérot, I. , J. Vignau , M. D'Herbomez , et al. 2007. “Evaluation of Bone Loss and Its Mechanisms in Anorexia Nervosa.” Calcified Tissue International 81, no. 3: 174–182. 10.1007/s00223-007-9038-9. 17668143

[erv70139-bib-0035] Lock, J. , and D. Le Grange . 2015. Treatment Manual for Anorexia Nervosa. A Family‐based Approach. Guilford Press.

[erv70139-bib-0036] Mainz, V. , M. Schulte‐Rüther , G. R. Fink , B. Herpertz‐Dahlmann , and K. Konrad . 2012. “Structural Brain Abnormalities in Adolescent Anorexia Nervosa Before and After Weight Recovery and Associated Hormonal Changes.” Psychosomatic Medicine 74, no. 6: 574–582. 10.1097/psy.0b013e31824ef10e. 22511729

[erv70139-bib-0037] Markevych, I. , T. Astell‐Burt , H. Altug , et al. 2022. “Residential Green Space and Age at Menarche in German and Australian Adolescent Girls: A Longitudinal Study.” International Journal of Hygiene and Environmental Health 240: 113917. 10.1016/j.ijheh.2021.113917. 35007986

[erv70139-bib-0038] Martinez, G. M. 2020. “Trends and Patterns in Menarche in the United States: 1995 Through 2013–2017.” National Health Statistics Reports, no. 146: 1–12. 33054923

[erv70139-bib-0039] Medical Management of Restrictive Eating Disorders in Adolescents and Young Adults . 2022. “Medical Management of Restrictive Eating Disorders in Adolescents and Young Adults.” Journal of Adolescent Health 71, no. 5: 648–654. 10.1016/j.jadohealth.2022.08.006. PMC10864000 36058805

[erv70139-bib-0040] Misra, M. , and A. Klibanski . 2014. “Endocrine Consequences of Anorexia Nervosa.” Lancet Diabetes & Endocrinology 2, no. 7: 581–592. 10.1016/s2213-8587(13)70180-3. PMC4133106 24731664

[erv70139-bib-0041] Misra, M. , R. Prabhakaran , K. K. Miller , et al. 2008. “Weight Gain and Restoration of Menses as Predictors of Bone Mineral Density Change in Adolescent Girls With Anorexia Nervosa‐1.” Journal of Clinical Endocrinology and Metabolism 93, no. 4: 1231–1237. 10.1210/jc.2007-1434. PMC2291495 18089702

[erv70139-bib-0042] Nadler, J. , C. U. Correll , D. Le Grange , E. C. Accurso , and V. Haas . 2022. “The Impact of Inpatient Multimodal Treatment or Family‐Based Treatment on Six‐Month Weight Outcomes in Youth With Anorexia Nervosa: A Naturalistic, Cross‐Continental Comparison.” Nutrients 14, no. 7: 1396. 10.3390/nu14071396. PMC9003203 35406009

[erv70139-bib-0043] NICE Guidelines . n.d. “Eating Disorders: Recognition and Treatment.” Accessed August 15, 2024. https://www.nice.org.uk/guidance/ng69.

[erv70139-bib-0044] Norris, M. L. , J. D. Hiebert , and D. K. Katzman . 2018. “Determining Treatment Goal Weights for Children and Adolescents With Anorexia Nervosa.” Paediatrics and Child Health 23, no. 8: 551–552. 10.1093/pch/pxy133. PMC6242059 31043839

[erv70139-bib-0045] Pitts, S. , E. Blood , A. Divasta , and C. M. Gordon . 2014. “Percentage Body Fat by Dual‐Energy X‐Ray Absorptiometry Is Associated With Menstrual Recovery in Adolescents With Anorexia Nervosa.” Journal of Adolescent Health 54, no. 6: 739–741. 10.1016/j.jadohealth.2013.12.033. PMC4035435 24613094

[erv70139-bib-0046] Pruccoli, J. , R. Pugliano , B. Pranzetti , and A. Parmeggiani . 2023. “Premenarchal Anorexia Nervosa: Clinical Features, Psychopharmacological Interventions, and Rehospitalization Analysis in a 1‐Year Follow‐Up, Controlled Study.” European Journal of Pediatrics 182, no. 6: 2855–2864. 10.1007/s00431-023-04960-y. PMC10257596 37052674

[erv70139-bib-0047] Ramraj, B. , and V. M. Subramanian . 2021. “V. G, Study on Age of Menarche Between Generations and the Factors Associated With It.” Clinical Epidemiology and Global Health 11: 100758. 10.1016/j.cegh.2021.100758.

[erv70139-bib-0048] Rastogi, R. , E. H. Sieke , A. Nahra , J. Sabik , and E. S. Rome . 2020. “Return of Menses in Previously Overweight Patients With Eating Disorders.” Journal of Pediatric and Adolescent Gynecology 33, no. 2: 133–138. 10.1016/j.jpag.2019.11.002. 31715368

[erv70139-bib-0049] Rosen, D. S. 2010. “Identification and Management of Eating Disorders in Children and Adolescents.” Pediatrics 126, no. 6: 1240–1253. 10.1542/peds.2010-2821. 21115584

[erv70139-bib-0050] Schaumberg, K. 2025. “TeenGrowth: Individualized Estimations of Weight‐Related Risk and Recovery Metrics for Young People With Eating Disorders.” International Journal of Eating Disorders 58, no. 4: 658–668. 10.1002/eat.24372. PMC11971021 39760235

[erv70139-bib-0051] Schlegl, S. , L. Vierl , D. R. Kolar , N. Dittmer , and U. Voderholzer . 2022. “Psychometric Properties of the Compulsive Exercise Test in a Large Sample of Female Adolescent and Adult Inpatients With Anorexia Nervosa and Bulimia Nervosa.” International Journal of Eating Disorders 55, no. 4: 494–504. 10.1002/eat.23694. 35199345

[erv70139-bib-0052] Sheehan, D. V. , Y. Lecrubier , K. H. Sheehan , et al. 1998. “The Mini‐International Neuropsychiatric Interview (M.I.N.I.): The Development and Validation of a Structured Diagnostic Psychiatric Interview for DSM‐IV and ICD‐10.” Supplement, Journal of Clinical Psychiatry 59, no. S20: 22–57: 22‐33;quiz 34‐57. 10.1037/t18597-000. 9881538

[erv70139-bib-0053] Solmi, M. , J. Radua , M. Olivola , et al. 2022. “Age at Onset of Mental Disorders Worldwide: Large‐Scale Meta‐Analysis of 192 Epidemiological Studies.” Molecular Psychiatry 27, no. 1: 281–295. 10.1038/s41380-021-01161-7. PMC8960395 34079068

[erv70139-bib-0054] Steinberg, D. M. , T. R. Perry , D. Freestone , M. Hellner , J. H. Baker , and C. Bohon . 2023. “Evaluating Differences in Setting Expected Body Weight for Children and Adolescents in Eating Disorder Treatment.” International Journal of Eating Disorders 56, no. 3: 595–603. 10.1002/eat.23868. 36458505

[erv70139-bib-0055] Swenne, I. 2004. “Weight Requirements for Return of Menstruations in Teenage Girls With Eating Disorders, Weight Loss and Secondary Amenorrhoea.” Acta Paediatrica 93, no. 11: 1449–1455. 10.1111/j.1651-2227.2004.tb02628.x. 15513571

[erv70139-bib-0056] van Eeden, A. E. , D. van Hoeken , J. M. T. Hendriksen , and H. W. Hoek . 2023. “Increase in Incidence of Anorexia Nervosa Among 10‐ to 14‐Year‐Old Girls: A Nationwide Study in the Netherlands Over Four Decades.” International Journal of Eating Disorders 56, no. 12: 2295–2303. 10.1002/eat.24064. 37773004

