# Supplementary material for: Resumption of Menses and Normalisation of Body Weight in Adolescents Undergoing Specialised Treatment for Anorexia Nervosa Inpatient or Outpatient Treatment
Source: Eur Eat Disord Rev. 2026 Jun 18;34(6):1515–27. doi: 10.1002/erv.70139 (PMC13656765; doi:10.1002/erv.70139)
Supplement: Supplementary file 1 [file ERV-34-1515-s001.docx]

**Supplemental Material for** **“Resumption of Menses and Normalization of Body Weight in Adolescents Undergoing Specialized Treatment for Anorexia Nervosa Inpatient or Outpatient Treatment“**

Supplemental Methods:

Patients for the current study were drawn from either one of two prospective, naturalistic IMT outcome studies [1], from a prospective FBT pilot study [2] or from a naturalistic FBT outcome study and were required to have both a baseline and 12FU assessment. In brief, the IMT studies [1] are two consecutive prospective, naturalistic, observational and longitudinal studies (still ongoing) aiming to assess clinical outcomes in youth who are hospitalized for AN. Eligible patients are admitted and treated for AN at the inpatient child and adolescent psychiatric units of an university hospital’s Department of Child and Adolescent Psychiatry, that is specialized in the treatment of eating disorders. Included were adolescents ages 8-17 years meeting the diagnostic criteria for either restrictive, binge-purge or atypical AN subtype according to ICD-10 [3] or DSM-V [4] and who were admitted between 10/2018 and 01/2024 with an age-and-sex adjusted BMI percentile <10.

In brief, for the FBT pilot [2] study and for the subsequent naturalistic FBT outcome study (still ongoing) patients were admitted between 01/2020 and 01/2024. Patients were recruited from the waiting lists of the same child and adolescent inpatient units specialized in the treatment of eating disorders mentioned above or families have contacted the FBT team directly. Inclusion criteria for the FBT arm were age 8-17 years, a diagnosis of AN of the subtypes restrictive, binge-purge an atypical AN, living within a distance to the study site that could be covered by car for face-to-face FBT sessions and knowledge of the German language, so that the therapy could be delivered in German. All FBT patients fulfilled the criteria associated with a recommendation for inpatient treatment according to the German S3-guidelines [5] operationalized as BMI <3rd percentile OR BMI <10th BMI percentile AND the presence of a significant co-morbid disorder OR rapid weight loss (>20% of body weight in the past 6 months) OR no weight gain despite ≥3 months of outpatient treatment. FBT patients were medically stable as defined by the Guidelines of the Society of Adolescent Health and Medicine [6]. These criteria include a %mBMI >75, daytime heart rate >50 bpm, blood pressure >80/50 mmHg and body temperature >35.6°C. When these criteria were not met, patients were medically stabilized either in the pediatric unit or in child and adolescent psychiatry wards. Exclusion criteria for FBT comprised existing child protection concerns, as well as risk of harm to self or others due to an acute psychiatric crisis that could not be managed in an outpatient setting.

References:

1. J. Nadler, C.U. Correll, D. Le Grange, E.C. Accurso, V. Haas, The Impact of Inpatient Multimodal Treatment or Family-Based Treatment on Six-Month Weight Outcomes in Youth with Anorexia Nervosa: A Naturalistic, Cross-Continental Comparison, Nutrients 14 (2022) 1396.

2. V. Haas, K. Wechsung, V. Kaiser, J. Schmidt, K. Raile, A. Busjahn, D. Le Grange, C.U. Correll, Comparing family-based treatment with inpatient treatment in youth with anorexia nervosa eligible for hospitalization: A 12-month feasibility study, The International journal of eating disorders 57 (2024) 388–399.

3. D.V. Sheehan, Y. Lecrubier, K.H. Sheehan, P. Amorim, J. Janavs, E. Weiller, T. Hergueta, R. Baker, G.C. Dunbar, The Mini-International Neuropsychiatric Interview (M.I.N.I.): the development and validation of a structured diagnostic psychiatric interview for DSM-IV and ICD-10, The Journal of clinical psychiatry 59 Suppl 20 (1998) 22-33;quiz 34-57.

4. A.P. Association, Diagnostic and Statistical Manual of Mental Disorders, American Psychiatric Association Publishing, 2022.

5. S. Herpertz, M. Fichter, B. Herpertz-Dahlmann, A. Hilbert, B. Tuschen-Caffier, S. Vocks, A. Zeeck, S3-Leitlinie Diagnostik und Behandlung der Essstörungen, Springer Berlin Heidelberg, Berlin, Heidelberg, 2019.

6. N.H. Golden, D.K. Katzman, R.E. Kreipe, S.L. Stevens, S.M. Sawyer, J. Rees, D. Nicholls, E.S. Rome, Eating disorders in adolescents: position paper of the Society for Adolescent Medicine, Journal of Adolescent Health 33 (2003) 496–503.

Supplemental Results:

| **Table S1.** Baseline characteristics of the total sample and of the analysed subgroups. | | | | | | | |
| --- | --- | --- | --- | --- | --- | --- | --- |
|  | **Total group**  **(*N* = 124)** | **12FU available**  **(*n* = 99)** | **12FU not available**  **(*n* = 25)** | ***p*-value (*n* = 99 vs. *n* = 25)** | **Adolescents** **with secondary amenorrhea at baseline (*n* = 82)** | | ***p*-value**  **(*n* = 41 vs. *n* = 41)** |
|  |  |  |  |  | **Completed menstrual questionnaire**  **(*n* = 41)** | **Did not complete menstrual questionnaire**  **(*n* = 41)** |  |
| Age (years), mean ± SD | 14.8 ± 1.6 | 14.8 ± 1.7 | 14.7 ± 1.4 | 0.814 | 14.9 ± 1.4 | 15.4 ± 1.4 | 0.101 |
| Height (cm), median (IQR) | 164.4 (160.0, 170.0) | 164.7 (159.0, 170.0) | 164.0 (160.3, 170.0) | 0.965 | 166.0 (161.0, 172.8) | 165.0 (162.0, 170.0) | 0.589 |
| Weight (kg), median (IQR) | 42.9 (39.7, 45.6) | 43.0 (39.7, 46.0) | 42.1 (39.6, 43.8) | 0.349 | 43.6 (41.1, 47.2) | 43.2 (41.4, 45.5) | 0.915 |
| BMI (kg/m²), median (IQR) | 15.6 (14.8, 16.5) | 15.6 (14.8, 16.5) | 15.5 (14.5, 16.4) | 0.423 | 15.9 (15.1, 16.4) | 16.0 (15.0, 16.6) | 0.770 |
| %mBMI median, (IQR) | 78.7 (74.3, 82.7) | 78.9 (74.3, 82.8) | 78.2 (73.5, 82.4) | 0.365 | 78.8 (75.5, 83.7) | 78.6 (74.1, 82.5) | 0.455 |
| BMI percentile, median (IQR) | 2.0 (0.0, 6.0) | 2.0 (0.0, 6.0) | 2.0 (0.0, 4.5) | 0.401 | 2.0 (0.5, 6.5) | 2.0 (0.0, 5.5) | 0.592 |
| Z-score, median (IQR) | -2.10 (-2.70, -1.60) | -2.04 (-2.68, -1.58) | -2.34 (-3.00, -1.72) | 0.134 | -2.10 (-2.73, -1.63) | -2.14 (-2.73, -1.74) | 0.683 |
| Premorbid weight (kg),  mean ± SD | 52.8 ± 9,4^‡^ | 52.7 ± 9.5^†^ | 53.6 ± 8.9^†^ | 0.654 | 55.3 ± 7.6 | 55.5 ± 8.1^‡^ | 0.884 |
| Weight suppression (kg),  mean ± SD | 10.3 ± 5.9^‡^ | 9.9 ± 5.9^†^ | 12.0 ± 5.78^†^ | 0.128 | 11.4 ± 5.7 | 11.9 ± 6.5^‡^ | 0.718 |
| Subtype, *N* (%) |  |  |  | 0.107 |  |  | 0.039* |
| Restrictive AN | 106 (85%) | 85 (86%) | 21 (84%) |  | 33 (80%) | 40 (98%) |  |
| Atypical AN | 10 (8%) | 6 (6%) | 4 (16%) |  | 4 (10%) | 1 (2%) |  |
| Binge-purge AN | 8 (6%) | 8 (8%) | 0 |  | 4 (10%) | 0 |  |
| Psychotropic medication, *n* (%) |  |  |  | 0.738 |  |  | 0.500 |
| No | 108 (87%) | 87 (88%) | 21 (84%) |  | 37 (90%) | 35 (85%) |  |
| Yes | 16 (13%) | 12 (12%) | 4 (16%) |  | 4 (10%) | 6 (14%) |  |
| Number of medications, median (IQR) | 0.0 (0.0, 0.0) | 0.0 (0.0, 0.0) | 0.0 (0.0, 0.0) | 0.577 | 0.0 (0.0, 0.0) | 0.0 (0.0, 0.0) | 0.502 |
| Medication type, *n* (%) |  |  |  |  |  |  |  |
| SSRI/SNRI | 12 (10%) | 8 (8%) | 4 (16%) | 0.259 | 3 (7%) | 4 (10%) | 1.000 |
| SGA | 5 (4%) | 5 (5%) | 0 | 0.582 | 1 (2%) | 2 (5%) | 1.000 |
| Tranquilizer | 1 (1%) | 0 | 1 (4%) | 0.202 | 0 | 0 | - |
| Comorbidities, *n* (%) |  |  |  | 0.968 |  |  | - |
| No | 69 (56%) | 55 (56%) | 14 (56%) |  | 22 (54%) | 22 (54%) |  |
| Yes | 55 (44%) | 44 (44%) | 11 (44%) |  | 19 (46%) | 19 (46%) |  |
| Number of comorbidities, median (IQR) | 0.0 (0.0,1.0) | 0.0 (0.0,1.0) | 0.0 (0.0,1.0) | 0.640 | 0.0 (0.0,1.0) | 0.0 (0.0,1.0) | 0.733 |
| Comorbidities type, *n* (%) |  |  |  |  |  |  |  |
| Depressive disorders | 37 (30%) | 30 (30%) | 7 (28%) | 0.822 | 18 (44%) | 8 (19%) | 0.018* |
| OCD | 16 (13%) | 14 (14%) | 2 (8%) | 0.523 | 3 (7%) | 9 (21%) | 0.116 |
| Anxiety disorders | 16 (13%) | 14 (14%) | 2 (8%) | 0.523 | 6 (15%) | 4 (10%) | 0.737 |
| Personality pathology | 7 (6%) | 5 (5%) | 2 (8%) | 0.628 | 1 (2%) | 2 (5%) | 1.000 |
| Other | 3 (2%) | 3 (3%) | 0 | 1.000 | 1 (2%) | 1 (2%) | 1.000 |
| CET, median (IQR) | 13.5 (10.1, 17.5)^‡^ | 13.3 (10.8, 17.3)^‡^ | 13.8 (6.6, 18.1) | 0.675 | 12.7 (8.1, 16.2) | 14.4 (11.4, 18.4)^‡^ | 0.134 |
| EDEQ |  |  |  |  |  |  |  |
| Global | 3.7 (2.1, 4.7)^†^ | 3.5 (2.0, 4.7)^†^ | 4.0 (2.2, 5.0) | 0.642 | 3.6 (2.0, 4.7) | 4.0 (2.3, 4.7)^†^ | 0.439 |
| Restraint | 3.4 (1.6, 4.8)^†^ | 3.4 (1.4, 4.8)^†^ | 3.4 (1.9, 5.1) | 0.357 | 3.4 (1.5, 4.8) | 3.6 (1.9, 5.4)^†^ | 0.269 |
| Eating concern | 2.8 (1.8, 4.0)^†^ | 2.8 (1.8, 4.0)^†^ | 3.1 (2.1, 4.0) | 0.801 | 2.4 (1.8, 4.1) | 3.2 (2.1, 4.0)^†^ | 0.401 |
| Weight concern | 3.4 (1.8, 5.2)^†^ | 3.4 (1.8, 5.2)^†^ | 4.3 (1.4, 5.3) | 0.621 | 3.4 (1.8, 5.2) | 3.6 (2.2, 5.3)^†^ | 0.795 |
| Shape concern | 4.4 (2.8, 5.6)^†^ | 4.4 (2.8, 5.6)^†^ | 4.4 (2.6, 5.6) | 0.985 | 4.3 (3.1, 5.6) | 4.9 (2.5, 5.6)^†^ | 0.590 |
| Menstruation, *n* (%) |  |  |  | 0.875 |  |  | - |
| Secondary amenorrhea | 82 (66%) | 66 (67%) | 16 (64%) |  | 41 (100%) | 41 (100%) |  |
| No menarche | 32 (26%) | 26 (26%) | 6 (24%) |  | 0 | 0 |  |
| Regular menses | 7 (6%) | 5 (5%) | 2 (8%) |  | 0 | 0 |  |
| Irregular menses | 0 | 0 | 0 |  | 0 | 0 |  |
| Contraceptive use < 3 months | 3 (2%) | 2 (2%) | 1 (4%) |  | 0 | 0 |  |
| Duration of treatment (days), median (IQR) | 156 (105, 217)^†^ | 166 (111, 238)^†^ | 114 (81, 160) | 0.003* | 161 (106, 210)^†^ | 139 (100, 216) | 0.727 |
| IMT | 123 (96, 165) | 125 (100, 168) | 122 (83, 158) | 0.518 | 142 (99, 183) | 123 (93, 163) | 0.620 |
| FBT | 213 (117, 334)^†^ | 237 (162, 354)^†^ | 112 (18, 180) | 0.003* | 189 (117, 318)^†^ | 221 (113, 243) | 0.723 |
| Treatment, *n* (%) |  |  |  | 0.054 |  |  | 0.325 |
| IMT | 68 (55%) | 50 (51%) | 18 (72%) |  | 18 (44%) | 24 (59%) |  |
| FBT | 56 (45%) | 49 (50%) | 7 (28%) |  | 23 (56%) | 17 (42%) |  |
| *Abbreviations: 12FU, 12-month follow-p; AN, anorexia nervosa; BMI, body mass index; CET,* *compulsive exercise test; cm, centimetre; EDEQ, eating disorder examination questionnaire; FBT, family-based treatment; IMT, inpatient multimodal treatment; kg, kilogram; %mBMI, percent median body mass index based on percentile curves by Kromeyer–Hausschild (Germany); OCD, obsessive-compulsive disorder; SGA, second-generation antipsychotics; SNRI, serotonin-norepinephrine reuptake inhibitor; SSRI, selective serotonin reuptake inhibitor; vs., versus.*  *^†^ Data from one patient were not available. ^‡^ Data from two patients were not available. Significant group differences: *p < 0.05.* | | | | | | | |

| **Table S2.** Comparisons of baseline characteristics of adolescents with secondary amenorrhea at baseline between those with and without regular menses at 12-month follow-up. | | | | | | | | |
| --- | --- | --- | --- | --- | --- | --- | --- | --- |
|  |  | | **Secondary amenorrhea at baseline (*N* = 66)** |  | **Menses at 12FU**  **(*n* = 35)** |  | **No menses at 12FU**  **(*n* = 31)** | ***p*-value** |
| Age (years), mean ± SD [range] |  | | 15.2 ± 1.4 [12.6 – 17.7] |  | 15.2 ± 1.1 [13.0 – 17.3] |  | 15.2 ± 1.6 [12.6 – 17.7] | 0.930 |
| Height (cm), median (IQR) |  | | 166.5 ± 6.4 [156.0, 182.0] |  | 166.0 ± 6.2 [165.0, 182.0] |  | 167.0 ± 6.7 [156.0, 180.0] | 0.528 |
| Weight (kg), mean ± SD [range] |  | | 43.9 ± 4.7 (29.7 – 56.0) |  | 44.9 ± 3.9 [36.0 – 56.0] |  | 42.7 ± 5.3 [29.9 – 52.6] | 0.056 |
| BMI (kg/m²), median (IQR) |  | | 16.1 (15.1, 16.5) |  | 16.4 (15.4, 16.8) |  | 15.2 (14.6, 16.2) | 0.001** |
| Premorbid weight(kg),  mean ± SD [range] |  | | 55.2 ± 7.7 [40.0 – 70.2] |  | 56.6 ± 7.5 [41.0 – 70.2] |  | 53.7 ± 7.6 [40.0 – 70.0] | 0.116 |
| Weight suppression (kg),  mean ± SD [range] |  | | 11.4 ± 6.0 [-4.7 – 26.6] |  | 11.8 ± 6.5 [0.6 – 26.6] |  | 11.0 ± 5.5 [-4.7 – 21.2] | 0.605 |
| %mBMI, median (IQR) |  | | 78.8 (74.4, 82.7) |  | 80.9 (76.7, 84.5) |  | 76.8 (73.3, 97.8) | 0.003** |
| BMI percentile, median (IQR) |  | | 2.0 (0.0, 6.0) |  | 3.0 (1.0, 8.0) |  | 1.0 (0.0, 3.0) | 0.009** |
| Z-score, median (IQR) |  | | -2.07 (-2.75, -1.70) |  | -1.91 (-2.39, -1.47) |  | -2.56 (-3.29, -1.85) | 0.005** |
| CET, median (IQR) |  | | 13.1 (10.7, 16.5) |  | 13.8 (11.9, 15.9) |  | 13.1 (9.3, 17.7) | 0.576 |
| EDEQ global, median (IQR) |  | | 3.7 (2.2, 4.7) |  | 3.8 (3.2, 4.7) |  | 3.1 (1.6, 4.7) | 0.129 |
| Subtype, *n* (%) |  | |  |  |  |  |  | 0.414 |
| Restrictive AN |  | | 58 (88%) |  | 29 (83%) |  | 29 (94%) |  |
| Atypical AN |  | | 4 (6%) |  | 3 (9%) |  | 1 (3%) |  |
| Binge-purge AN |  | | 4 (6%) |  | 3 (9%) |  | 1 (3%) |  |
| Psychotropic medication, *n* (%) |  | |  |  |  |  |  | 0.265 |
| No |  | | 58 (88%) |  | 29 (83%) |  | 29 (94%) |  |
| Yes |  | | 8 (12%) |  | 6 (17%) |  | 2 (6%) |  |
| Number of medications, median (IQR) |  | | 0.0 (0.0, 0.0) |  | 0.0 (0.0, 0.0) |  | 0.0 (0.0, 0.0) | 0.187 |
| Medication type, *n* (%) |  | |  |  |  |  |  |  |
| SSRI/SNRI |  | | 5 (8%) |  | 3 (9%) |  | 2 (6%) | 1.000 |
| SGA |  | | 3 (5%) |  | 3 (9%) |  | 0 | 0.241 |
| Tranquilizer |  | | 0 |  | 0 |  | 0 | - |
| Other |  | | 0 |  | 0 |  | 0 | - |
| Comorbidities, *n* (%) |  | |  |  |  |  |  | 0.300 |
| No |  | | 36 (55%) |  | 17 (49%) |  | 19 (61%) |  |
| Yes |  | | 30 (45%) |  | 18 (51%) |  | 12 (39%) |  |
| Number of comorbidities, median (IQR) |  | | 0.0 (0.0, 1.0) |  | 0.0 (1.0, 1.0) |  | 0.0 (0.0, 1.0) | 0.188 |
| Comorbidities type, *n* (%) |  | |  |  |  |  |  |  |
| Depressive disorders |  | | 19 (29%) |  | 10 (29%) |  | 9 (29%) | 0.967 |
| OCD |  | | 11 (17%) |  | 7 (20%) |  | 4 (13%) | 0.521 |
| Anxiety disorders |  | | 7 (11%) |  | 6 (17%) |  | 1 (3%) | 0.110 |
| Personality pathology |  | | 2 (3%) |  | 1 (3%) |  | 1 (3%) | 1.000 |
| Other | |  | 2 (3%) |  | 2 (6%) |  | 0 | 0.494 |
| Duration of treatment (days), median (IQR) | |  | 164 (106, 234)^†^ |  | 168 (104, 238)^†^ |  | 164 (115, 237) | 0.823 |
| IMT | |  | 121 (99, 168) |  | 213 (101, 173) |  | 135 (95, 164) | 0.937 |
| FBT |  | | 211 (132, 315)^†^ |  | 211 (115, 317)^†^ |  | 231 (160, 366) | 0.732 |
| Treatment, *n* (%) |  | |  |  |  |  |  | 0.828 |
| IMT |  | | 35 (53%) |  | 19 (54%) |  | 16 (52%) |  |
| FBT |  | | 31 (47%) |  | 16 (46%) |  | 15 (48%) |  |

*Abbreviations: 12FU, 12-month follow-up; AN, anorexia nervosa; BMI, body mass index; CET,* *compulsive exercise test; cm, centimetre; EDEQ, eating disorder examination questionnaire; FBT, family-based treatment; IMT, inpatient multimodal treatment; kg, kilogram; %mBMI, percent median body mass index based on percentile curves by Kromeyer–Hausschild (Germany); OCD, obsessive-compulsive disorder; SGA, second-generation antipsychotics; SNRI, serotonin-norepinephrine reuptake inhibitor; SSRI, selective serotonin reuptake inhibitor.*

*^†^ Data from one patient were not available. Significant group differences: **p < 0.01.*

| **Table S3.** Comparisons of weight parameters from baseline to 6- and 12-month follow-up between adolescents with secondary amenorrhea at baseline with or without regular menses at 12-month follow-up. | | | | | | | | |
| --- | --- | --- | --- | --- | --- | --- | --- | --- |
|  |  |  | **Secondary amenorrhea at baseline (*N* = 66)** |  | **Menses at 12FU**  **(*n* = 35)** |  | **No menses at 12FU**  **(*n* = 31)** | ***p*-value** |
| Height (cm), median (IQR) | BL  6FU  12FU  Δ (12FU - BL) |  | 166.5 ± 6.4 [156.0 –182.0] 166.5 ± 6.2 [155.0 – 181.8]  167.0 ± 6.1 [156.0 – 181.0]  0.5 ± 1.7 [-0.3 – 0.6] |  | 166.0 ± 6.2 [165.0 – 182.0] 166.2 ± 6.0 [156.0 – 181.8]  166.6 ± 5.8 [158.0 – 181.0]  0.6 ± 1.2 [-2.0 – 4.0] |  | 167.0 ± 6.7 [156.0 – 180.0]  166.8 ± 6.4 [155.0 – 178.0)  167.4 ± 6.7 [156.0 – 180.0]  0.4 ± 2.1 [-3.0 – 6.0] | 0.528  0.714  0.610  0.605 |
| Weight (kg), median (IQR) | BL  6FU  12FU  Δ (12FU - BL) |  | 43.9 ± 4.7 [29.7 – 56.0]  51.4 ± 5.4 [36.6 – 69.0]  51.9 ± 6.3 [38.8 – 69.0)  8.0 ± 5.4 [-3.4 – 24.0] |  | 44.9 ± 3.9 [36.0 – 56.0]  51.9 ± 4.2 [44.6 – 60.0]  53.9 ± 5.9 [41.9 – 69.0]  9.0 ± 5.1 [0.7 – 24.0] |  | 42.7 ± 5.3 [29.9 – 52.6]  50.7 ± 6.7 [36.6 – 69.0]  49.6 ± 5.6 [38.8 – 63.4]  6.9 ± 5.7 (-3.4 – 18.1) | 0.056  0.410  0.004**  0.110 |
| BMI (kg/m^2^), median (IQR) | BL  6FU  12FU  Δ (12FU - BL) |  | 16.1 (15.1, 16.5)  18.3 (17.5, 19.5)  18.5 (17.4, 19.3)  2.5 (1.4, 4.1) |  | 16.4 (15.4, 16.8)  18.8 (17.7, 19.8)  19.1 (18.1, 20.0)  2.6 (1.7, 4.0) |  | 15.2 (14.6, 16.2)  17.8 (17.3, 18.9)  17.6 (16.7, 18.6)  2.3 (1.3, 4.2) | 0.001**  0.069  < 0.001***  0.286 |
| %mBMI,  median (IQR) | BL  6FU  12FU  Δ (12FU - BL) |  | 78.8 (74.4, 82.7)  89.6 (85.6, 95.0)  90.2 (85.2, 96.8)  11.0 (5.1, 19.0) |  | 80.9 (76.7, 84.5)  92.7 (86.5, 97.5)  92.4 (88.9, 98.3)  11.1 (6.4, 18.4) |  | 76.8 (73.3, 97.8)  87.5 (85.3, 92.2)  87.6 (82.2, 92.3)  9.8 (4.1, 19.6) | 0.003**  0.042*  0.003**  0.355 |
| BMI percentile, median (IQR) | BL  6FU  12FU  Δ (12FU - BL) |  | 2.0 (0.0, 6.0)  18.0 (11.0, 34.0)  19.0 (8.0, 36.8)  14.5 (5.0, 34.3) |  | 3.0 (1.0, 8.0)  27.0 (12.0, 43.0)  27.0 (17.0, 44.0)  22.0 (10.0, 40.0) |  | 1.0 (0.0, 3.0)  12.5 (8.5, 26.5)  11.0 (4.0, 20.0)  10.0 (2.0, 18.0) | 0.009**  0.034*  0.001**  0.007** |
| Z-score, median (IQR) | BL  6FU  12FU  Δ (12FU - BL) |  | -2.07 (-2.75, -1.70)  -0.90 (-1.34, -0.43)  -0.84 (-1.65, -0.31)  1.39 (0.86, 1.97) |  | -1.91 (-2.39, -1.47)  -0.60 (-1.19, -0.31)  -0.61 (-1.02, 0.01)  1.27 (0.84, 2.06) |  | -2.56 (-3.29, -1.85)  -1.10 (-1.44, -0.60)  -1.40 (-1.78, -0.80)  1.42 (0.60, 1.85) | 0.005**  0.026*  < 0.001***  0.615 |
| *Abbreviations: 12FU, 12-month follow-up; BL, baseline; BMI, body mass index, centimetre; IQR, interquartile range; kg, kilogram; %mBMI, percent median body mass index based on percentile curves by Kromeyer–Hausschild (Germany); Δ, delta.*  *Note: At 6FU data from three patients in the group ‘no menses at 12FU’ were not available. Significant group differences: *p < 0.05, **p < 0.01, ***p < 0.001.* | | | | | | | | |

|  | **Table S4.** Comparisons of compulsive exercise and eating disorder psychopathology at baseline from 6- and 12-month follow-up between adolescents with secondary amenorrhea at baseline with or without regular menses at 12-month follow-up. | | | | | | | | |
| --- | --- | --- | --- | --- | --- | --- | --- | --- | --- |
|  |  |  |  | **Secondary amenorrhea at baseline (*N* = 66)** |  | **Menses at 12FU**  **(*n* = 35)** |  | **No menses at 12FU**  **(*n* = 31)** | ***p*-value** |
|  | CET, *n* (%) | BL  6FU  12FU  Δ (12FU - BL) |  | 13.2 (10.7, 16.5)  11.1 (6.8, 14.3)  10.9 (6.3, 14.8)  -3.0 (-5.1, 2.4) |  | 13.8 (11.9, 15.9)  9.6 (6.4, 13.8)  9.5 (6.0, 13.6)  -3.6 (-6.9, 1.4) |  | 13.1 (9.3, 17.7)  11.7 (7.8, 15.6)  11.8 (7.5, 16.5)  -2.2 (-4.7, 2.6) | 0.576  0.168  0.328  0.323 |
|  | EDEQ global,  *n* (%) | BL  6FU  12FU  Δ (12FU - BL) |  | 3.7 (2.2, 4.7)  2.0 (1.0, 3.4)  1.8 (0.9, 4.2)  -1.0 (-2.2, -0.1) |  | 3.8 (3.2, 4.7)  2.1 (1.1, 3.8)  1.8 (1.0, 4.2)  -1.1 (-2.4, -0.3) |  | 3.1 (1.6, 4.7)  1.8 (0.9, 3.0)  1.9 (0.8, 4.1)  -0.7 (-1.8, 0.4) | 0.129  0.247  0.664  0.204 |
|  | EDEQ restraint, *n* (%) | BL  6FU  12FU  Δ (12FU - BL) |  | 3.4 (1.4, 5.0)  1.2 (0.4, 2.8)  1.2 (0.4, 3.8)  -0.9 (-2.8, 0.3) |  | 3.6 (2.2, 5.0)  1.0 (0.6, 2.8)  1.0 (0.4, 4.3)  -1.1 (-3.3, 0.0) |  | 3.0 (1.2, 4.8)  1.4 (0.3, 2.8)  1.4 (0.6, 3.6)  -0.7 (-2.0, 0.4) | 0.361  0.860  0.651  0.393 |
|  | EDEQ eating concern, *n* (%) | BL  6FU  12FU  Δ (12FU - BL) |  | 2.7 (1.8, 4.1)  1.4 (0.4, 2.4)  1.2 (0.6, 4.6)  -0.8 (-1.8, -0.2) |  | 3.4 (2.2, 4.2)  1.4 (0.6, 2.9)  1.2 (0.5, 3.4)  -1.0 (-2.0, -0.3) |  | 2.4 (1.6, 3.8)  1.2 (0.4, 2.2)  1.3 (0.6, 2.6)  -0.6 (-1.8, 0.0) | 0.166  0.408  0.954  0.173 |
|  | EDEQ weight concern, *n* (%) | BL  6FU  12FU  Δ (12FU - BL) |  | 3.5 (2.1, 5.2)  1.9 (0.8, 3.8)  2.0 (0.8, 4.6)  -1.0 (-2.4, 0.0) |  | 3.8 (3.0, 5.2)  2.2 (1.1, 4.2)  2.3 (1.1, 4.6)  -1.4 (-2.6, 0.2) |  | 2.8 (1.6, 5.2)  1.6 (0.6, 2.6)  1.8 (0.6, 4.5)  -0.6 (-1.8, 0.0) | 0.129  0.059  0.676  0.163 |
|  | EDEQ shape concern, *n* (%) | BL  6FU  12FU  Δ (12FU - BL) |  | 4.6 (3.2, 5.7)  2.9 (1.7, 5.0)  2.6 (1.4, 5,1)  -0.9 (-2.7, 0.0) |  | 4.9 (3.9, 5.6)  3.4 (1.9, 5.2)  2.4 (1.4, 5,1)  -1.4 (-3.1, 0.0) |  | 4.2 (2.3, 5.8)  2.9 (1.0, 4.6)  2.6 (1.0, 5,2)  -0.4 (-2.2, 0.1) | 0.313  0.146  0.780  0.119 |
| *Abbreviations: 6FU, 6-month follow-up; 12FU, 12-month follow-up; BL, baseline; CET, compulsive exercise test; EDEQ, eating disorder examination questionnaire; kg, kilogram; Δ, delta.*  *Note: At 6FU data from three patients in the group ‘no menses at 12FU’ were not available. Significant group differences: *p < 0.05.* | | | | | | | | | |

| **Table S5**. Multivariable stepwise backward logistic regression model for menses at 12-month follow-up in adolescents with secondary amenorrhea at baseline. *N* = 66. Cox & Snell R² 0.231 Nagelkerkes R² 0.308. Area under the curve = 0.777. | | | | | |
| --- | --- | --- | --- | --- | --- |
| **Independent predictors for menses at 12FU** | **Regression coefficient B** | **SE** | **Odds Ratio** | ***p*-value** | **95% CI** |
| BL %mBMI | 0.128 | 0.054 | 1.137 | 0.018* | 1.022 – 1.264 |
| 12FU %mBMI | 0.097 | 0.040 | 1.102 | 0.016* | 1.018 – 1.192 |
| *Abbreviations: 12FU, 12-month follow-up; B, regression coefficient; BL, baseline; CI, confidence interval; %mBMI, percentage of the median body mass index based on percentile curves by Kromeyer–Hausschild (Germany);* *SE, standard error.*  *Significant group differences: *p < 0.05* | | | | | |

| **Table S6.** Comparisons of baseline characteristics of adolescents without menarche at baseline between those with and without regular menses at 12-month follow-up. | | | | | | | | |
| --- | --- | --- | --- | --- | --- | --- | --- | --- |
|  |  | | **No menarche at baseline (*N* = 26)** |  | **Menses at 12FU**  **(*n* = 5)** |  | **No menses at 12FU**  **(*n* = 21)** | ***p*-value** |
| Age (years), mean ± SD |  | | 13.6 ± 1.8 |  | 15.0 ± 1.0 |  | 13.2 ± 1.8 | 0.053 |
| Height (cm), mean ± SD |  | | 157.7 ± 1.1 |  | 168.5 ± 1.2 |  | 155.1 ± 9.7 | 0.013* |
| Weight (kg), mean ± SD |  | | 37.7 ± 6.8 |  | 43.0 ± 4.7 |  | 36.5 ± 6.7 | 0.052 |
| BMI (kg/m²), mean ± SD |  | | 15.0 ± 1.1 |  | 15.1 ± 0.5 |  | 15.0 ± 1.3 | 0.839 |
| Premorbid weight (kg),  mean ± SD |  | | 44.8 ± 9.5^†^ |  | 52.0 ± 6.0 |  | 43.0 ± 9.5^†^ | 0.056 |
| Weight suppression (kg),  mean ± SD |  | | 7.1 ± 4.3^†^ |  | 9.0 ± 4.8 |  | 6.6 ± 4.2^†^ | 0.280 |
| %mBMI, mean ± SD |  | | 78.1 ± 5.5 |  | 75.3 ± 4.9 |  | 78.8 ± 5.7 | 0.215 |
| BMI percentile, median (IQR) |  | | 2.0 (0.0, 6.0) |  | 0.0 (1.0, 3.0) |  | 3.0 (1.0, 6.0) | 0.034* |
| Z-score, median (IQR) |  | | -2.10 (-2.67, -1.55) |  | -2.68 (-2.83, -2.11) |  | -1.99 (-2.53, -1.54) | 0.091 |
| CET, mean ± SD |  | | 14.1 ± 5.1^†^ |  | 16.0 ± 3.1 |  | 13.6 ± 5.4^†^ | 0.354 |
| EDEQ global, median (IQR) |  | | 3.3 (2.1, 4.6)^†^ |  | 3.8 (3.2, 4.7) |  | 3.1 (1.6, 4.7)^†^ | 0.112 |
| Subtype, *n* (%) |  | |  |  |  |  |  | 0.773 |
| Restrictive AN |  | | 24 (92%) |  | 5 (100%) |  | 19 (90%) |  |
| Atypical AN |  | | 1 (4%) |  | 0 |  | 1 (5%) |  |
| Binge-purge AN |  | | 1 (4%) |  | 0 |  | 1 (5%) |  |
| Psychotropic medication, *n* (%) |  | |  |  |  |  |  | 0.345 |
| No |  | | 24 (92%) |  | 5 (100%) |  | 19 (90%) |  |
| Yes |  | | 2 (8%) |  | 0 |  | 2 (10%) |  |
| Number of medications, median (IQR) |  | | 0.0 (0.0, 0.0) |  | 0.0 (0.0, 0.0) |  | 0.0 (0.0, 0.0) | 0.753 |
| Comorbidities, *n* (%) |  | |  |  |  |  |  | 0.055 |
| No |  | | 16 (62%) |  | 1 (20%) |  | 15 (71%) |  |
| Yes |  | | 10 (38%) |  | 4 (80%) |  | 6 (29%) |  |
| Number of comorbidities, median (IQR) |  | | 0.0 (0.0, 1.0) |  | 1.0 (0.5, 2,5) |  | 0.0 (0.0, 1.0) | 0.079 |
| Comorbidities type, *n* (%) |  | |  |  |  |  |  |  |
| Depressive disorders |  | | 7 (27%) |  | 3 (60%) |  | 4 (19%) | 0.101 |
| OCD |  | | 3 (12%) |  | 1 (20%) |  | 2 (10%) | 0.488 |
| Anxiety disorders |  | | 4 (15%) |  | 2 (40%) |  | 2 (10%) | 0.155 |
| Personality pathology |  | | 2 (8%) |  | 1 (20%) |  | 1 (5%) | 0.354 |
| Other |  | | 1 (4%) |  | 0 |  | 1 (5%) | - |
| Duration of treatment (days), median (IQR) | |  | 168 (118, 295) |  | 184 (133, 253) |  | 167 (115, 329) | 0.850 |
| IMT | |  | 138 (115, 172) |  | 147 (119, -) |  | 128 (114, 168 |  |
| FBT | |  | 298 (179, 362) |  | 253 (213, -) |  | 329 (154, 368) |  |
| Treatment, *n* (%) | |  |  |  |  |  |  | 1.000 |
| IMT | |  | 14 (54%) |  | 3 (60%) |  | 11 (52%) |  |
| FBT |  | | 12 (46%) |  | 2 (40%) |  | 10 (48%) |  |
| *Abbreviations: 12FU, 12-month follow-up; AN, anorexia nervosa; BMI, body mass index; CET,* *compulsive exercise test; cm, centimetre; EDEQ, eating disorder examination questionnaire; FBT, family-based treatment; IMT, inpatient multimodal treatment; IQR, interquartile range; kg, kilogram; %mBMI, percent median body mass index based on percentile curves by Kromeyer–Hausschild (Germany); OCD, obsessive-compulsive disorder; SGA, second-generation antipsychotics; SNRI, serotonin-norepinephrine reuptake inhibitor; SSRI, selective serotonin reuptake inhibitor.*  *^†^ Data from one patient were not available. Significant group differences: *p < 0.05.* | | | | | | | | |

| **Table S7.** Comparisons of weight parameters from baseline to 6- and 12-month follow-up between adolescents without menarche at baseline with or without regular menses at 12-month follow-up. | | | | | | | | |
| --- | --- | --- | --- | --- | --- | --- | --- | --- |
|  |  |  | **No menarche at baseline**  **(*N* = 26)** |  | **Menses at 12FU**  **(*n* = 5)** |  | **No menses at 12FU**  **(*n* = 21)** | ***p*-value** |
| Height (cm), median (IQR) | BL  6FU  12FU  Δ (12FU - BL) |  | 157.7 ± 1.1  158.8 ± 10.4  160.3 ± 9.8  2.6 ± 2.8 |  | 168.5 ± 1.2  169.1 ± 9.7  169.7 ± 8.6  1.1 ± 2.6 |  | 155.1 ± 9.7  156.3 ± 9.1  158.0 ± 8.9  2.9 ± 2.8 | 0.013*  0.010*  0.014*  0.198 |
| Weight (kg), median (IQR) | BL  6FU  12FU  Δ (12FU - BL) |  | 37.7 ± 6.8  44.7 ± 8.1  46.8 ± 8.2  9.1 ± 3.2 |  | 43.0 ± 4.7  52.5 ± 6.1  53.5 ± 3.9  10.4 ± 1.9 |  | 36.5 ± 6.7  42.8 ± 7.4  45.3 ± 8.2  8.8 ± 3.3 | 0.052  0.013*  0.041*  0.309 |
| BMI (kg/m^2^), median (IQR) | BL  6FU  12FU  Δ (12FU - BL) |  | 15.0 ± 1.1  17.5 ±1.4  18.1 ±1.8  3.1 ± 1.2 |  | 15.1 ± 0.5  18.3 ± 0.7  18.6 ± 0.9  3.5 ± 0.6 |  | 15.0 ± 1.3  17.3 ± 1.4  18.0 ± 1.9  3.0 ± 1.3 | 0.839  0.147  0.508  0.421 |
| %mBMI,  median (IQR) | BL  6FU  12FU  Δ (12FU - BL) |  | 78.1 ± 5.5  89.1 ±6.1  91.6 ± 8.1  13.5 ± 5.6 |  | 75.3 ± 4.9  89.9 ± 4.5  90.4 ± 6.2  15.1 ± 2.7 |  | 78.8 ± 5.7  89.0 ± 6.6  91.8 ± 8.6  13.1 ± 6.0 | 0.215  0.768  0.734  0.470 |
| BMI percentile, median (IQR) | BL  6FU  12FU  Δ (12FU - BL) |  | 2.0 (0.0, 6.0)  19.5 (12.8, 26.8)  23.5 (13.8, 31.8)  17.5 (12.8, 28.8) |  | 0.0 (0.0, 3.0)  22.0 (12.5, 28.0)  15.0 (10.5, 36.5)  15.0 (10.5, 35.5) |  | 3.0 (1.0, 6.0)  19.0 (12.5, 28.5)  25.0 (14.5, 34.5)  18.0 (13.0, 31.5) | 0.034*  0.705  0.200  0.527 |
| Z-score, median (IQR) | BL  6FU  12FU  Δ (12FU - BL) |  | -2.10 (-2.67, -1.55)  0.84 (-1.23, -0.57)  -0.73 (-1.08, -0.52)  1.46 (0.93, 1.87) |  | -2.68 (-2.83, -2.11)  -0.69 (-1.25, -0.55)  -1.02 (-1.23, -0.25)  1.79 (1.53, 1.87) |  | -1.99 (-2.53, -1.54)  -0.87 (-1.28, -0.56)  -0.67 (-1.06, -0.44)  1.44 (0.73, 1.87) | 0.091  0.659  0.374  0.224 |
| *Abbreviations: 6FU, 6-month follow-up; 12FU, 12-month follow-up; BL, baseline; BMI, body mass index; cm, centimeter; IQR, interquartile range; kg, kilogram; %mBMI, percent median body mass index based on percentile curves by Kromeyer–Hausschild (Germany); Δ, delta.*  *Significant group differences: *p < 0.05.* | | | | | | | | |

| **Table S8.** Comparisons of compulsive exercise and eating disorder psychopathology from baseline to 6- and 12-month follow-up between adolescents without menarche at baseline with or without regular menses at 12-month follow-up. | | | | | | | | |
| --- | --- | --- | --- | --- | --- | --- | --- | --- |
|  |  |  | **No menarche at baseline**  **(*N* = 25)** |  | **Menses at 12FU**  **(*n* = 5)** |  | **No menses at 12FU**  **(*n* = 20)** | ***p*-value** |
| CET, *n* (%) | BL  6FU  12FU  Δ (12FU - BL) |  | 14.1 ± 5.1  10.7 ± 5.1  9.1 ± 5.0  -4.8 ± 5.4 |  | 16.0 ± 3.1  14.2 ± 3.8  14.5 ± 3.3  -1.5 ± 2.6 |  | 13.6 ± 5.4  9.8 ± 5.1  7.6 ± 4.4  -5.8 ± 5.7 | 0.354  0.086  0.004**  0.123 |
| EDEQ global,  *n* (%) | BL  6FU  12FU  Δ (12FU - BL) |  | 3.3 (2.1, 4.6)  1.3 (0.8, 3.2)  1.3 (0.3, 3.1)  -1.6 (-2.6, -0.3) |  | 4.6 (2.8, 4.9)  5.0 (3.4, 5.8)  3.3 (2.0, 5.1)  -1.3 (-1.6, -1.0) |  | 3.0 (1.9, 4.5)  1.1 (0.6, 2.5)  0.9 (0.2, 1.9)  -1.8 (-3.5, -0.5) | 0.112  0.009**  0.009**  0.120 |
| EDEQ restraint,  *n* (%) | BL  6FU  12FU  Δ (12FU - BL) |  | 3.6 (1.4, 4.7)  1.0 (0.2, 2.2)  1.0 (0.2, 2.4)  -1.3 (-3.3, -0.4) |  | 4.0 (2.3, 4.8)  4.0 (1.5, 4.5)  4.2 (1.3, 5.2)  -1.0 (-1.4, 1.4) |  | 3.4 (1.3, 4.8)  0.7 (0.2, 1.3)  0.6 (0.0, 1.9)  -1.6 (-3.5, -0.5) | 0.575  0.012*  0.019*  0.120 |
| EDEQ eating concern, *n* (%) | BL  6FU  12FU  Δ (12FU - BL) |  | 2.8 (1.7, 3.6)  1.1 (80.4, 2.4)  0.8 (0.2, 2.2)  -1.4 (-2.3, -0.6) |  | 4.0 (2.9, 5.1)  2.4 (1.8, 3.2)  2.8 (1.3, 3.8)  -1.4 (-2.9, 0.1) |  | 2.7 (1.4, 4.8)  0.6 (0.4, 2.2)  0.7 (0.2, 1.9)  -1.4 (-2.1, -0.5) | 0.015*  0.030*  0.019*  0.880 |
| EDEQ weight concern, *n* (%) | BL  6FU  12FU  Δ (12FU - BL) |  | 2.6 (1.6, 5.0)  1.3 (0.6, 3.1)  1.6 (0.4, 3.4)  -0.7 (-2.3, 0.2) |  | 3.4 (2.1, 5.3)  2.6 (1.7, 4.8)  3.2 (1.5, 5.6)  0.0 (-1.6, 1.2) |  | 2.5 (1.4, 4.8)  1.0 (0.6, 2.4)  1.3 (0.2, 2.8)  -1.0 (-2.9, -0.2) | 0.488  0.053  0.067  0.283 |
| EDEQ shape concern, *n* (%) | BL  6FU  12FU  Δ (12FU - BL) |  | 3.9 (2.8, 5.5)  1.9 (1.0, 4.6)  1.6 (0.8, 4.3)  -1.4 (-2.7, -0.2) |  | 5.0 (3.4, 4.5)  5.3 (3.6, 5.8)  4.6 (2.9, 5.9)  -0.8 (-1.5, 1.3) |  | 3.7 (2.3, 5.2)  1.6 (0.9, 1.8)  1.3 (0.3, 3.2)  -1.5 (-2.9, -0.2) | 0.216  0.009**  0.009**  0.249 |
| *Abbreviations: 6FU, 6-month follow-up; 12FU, 12-month follow-up; BL, baseline; CET, compulsive exercise test; EDEQ, eating disorder examination questionnaire; kg, kilogram; Δ, delta.* *Note: At 6FU data from one patient in the group ‘no menses at 12FU’ were not available. At 12FU data from two patients in the group ‘no menses at 12FU’ were not available. The difference Δ (12FU - BL) was calculated for 17 patients in the group ‘no menses at 12FU’. Significant group differences: *p < 0.05, **p < 0.01.* | | | | | | | | |
